# Supplementary material for: Improving risk stratification and detection of early HCC using ultrasound-based deep learning models
Source: JHEP Rep. 2025 Jul 5;7(10):101510. doi: 10.1016/j.jhepr.2025.101510 (PMC12448012; doi:10.1016/j.jhepr.2025.101510)
Supplement: Multimedia component 1 [file mmc1.pdf]

Improving risk stratification and detection of early HCC using ultrasound-based deep learning models

Jérémy Dana, Adrien Meyer, Anita Paisant, Agnès Rode, Riccardo Sartoris, Olivier Séror, Christophe Cassinotto, Laurent Milot, Jules Grégory, Jules Cœur, Jérôme Lebigot, Valentina Schembri, François Villeret, Armelle-Natsuo Takeda, Maxime Ronot, Valérie Vilgrain, Thomas F Baumert, Benoit Gallix, Nicolas Padoy, Pierre Nahon

Table of contents

Supplementary methods……………………………………………………………... 2

Fig. S1……………………………………………………………………………….. 3

Fig. S2……………………………………………………………………………….. 3

Table S1……………………………………………………………………………...4

Table S2……………………………………………………………………………...4

Table S3……………………………………………………………………………...4

Table S4……………………………………………………………………………...5

Supplementary methods

Performance in Simulated Low-Prevalence Setting

Considering the diagnostic performance of the STARHE-RISK model in the testing set and the estimated real-life prevalence of HCC (3%), the simulated positive and negative predictive values are 7.4% and 98.8% for STARHE-RISK, 4.7% and 98.7% for FASTRAK, 11.4% and 98.5% for STARHE-RISK combined with FASTRAK, respectively. Regarding STARHE-DETECT, the simulated positive and negative predictive values were 10.5% and 98.8%, respectively.

Description of models' architecture for STARHE-RISK and STARHE-DETECT models

The C3D model is a 3D convolutional neural network designed for video input. It processes video clips using eight 3×3×3 convolutional layers with ReLU activation, interspersed with five 3D max-pooling layers. The output is then passed through two fully connected layers of 4,096 units each, followed by a softmax classifier for final prediction.

RTMDet is a one-stage, anchor-free object detector optimized for real-time performance. It strikes a balance between speed and accuracy using a streamlined CSP-Darknet backbone and a neck composed of large-kernel depth-wise convolutions to enhance feature representation. A two-layer regression head predicts bounding boxes, while a linear classification head assigns class labels. During training, dynamic soft-label assignment is used to address class imbalance effectively.

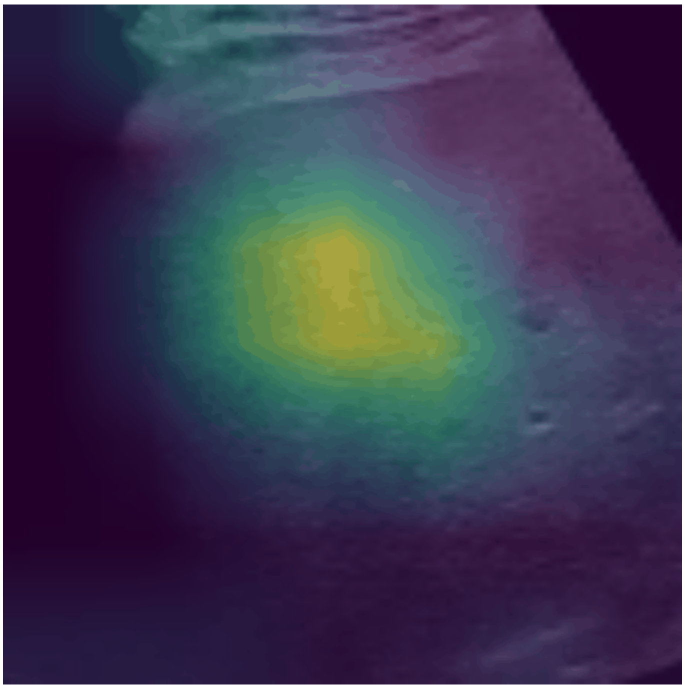

Fig. S1 – Example of Grad-CAM++ explainability map for the STARHE-RISK model demonstrating that the region of interest lies in the liver parenchyma.

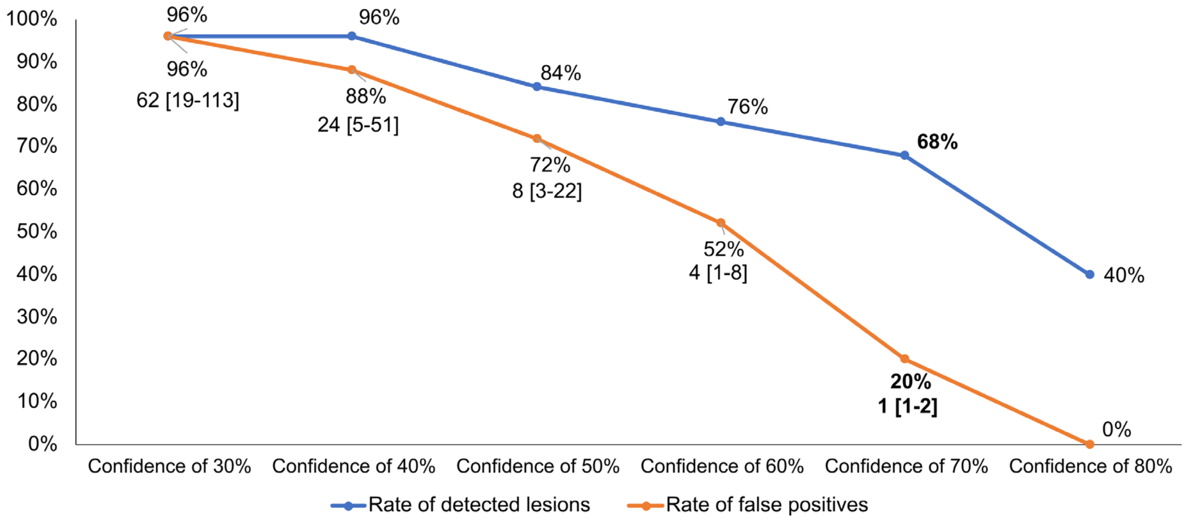

Fig. S2 – Rates of detected HCC nodules and false positives (with the median number of false positives and interquartile range per video) by the deep learning detection model on the 25 B-mode HCC ultrasound cine clips across different confidence levels in the predicted bounding box. A confidence level of 70% appeared to be the most clinically relevant as it achieved good performances with a rate of detected lesions of 68% (17/25).

	Model and hyperparameters	Final model
**Batch sizes**	2, 4, 8, 16, 32	4
**Learning rate**	0.000005, 0.00001, 0.000015, 0.00002, 0.000025, 0.00003, 0.0000375, 0.00005, 0.0001, 0.0016	45 epochs with learning rate of 0.00002 (divided by 10 at 20 and 40 epochs)
**Optimizer**	SGD, AdamW	SGD
**Input/output size**	128x128, 256x256	128x128
**Transfer learning**	All except prediction head	All except prediction head
**Models**	MViT, C3D, I3D	C3D

Table S1 - Model and hyperparameters selection for STARHE-RISK model.

	Model and hyperparameters	Final model
**Batch sizes**	2, 4, 8, 16	8
**Learning rate**	0.0008, 0.0006, 0.0004, 0.0002, 0.0001, 0.001, 0.002, 0.005	45 epochs with learning rate of 0.0002 (divided by 10 at 20 and 40 epochs)
**Frozen stages**	-1, 0, 1	-1 (none)
**Batch requires grads**	True, False	True
**Transfer learning**	All except prediction head	All except prediction head
**Models**	Faster-RCNN, DINO-DETR, RTMDet	RTMDet

Table S2 - Model and hyperparameters selection for STARHE-DETECT model

	Sensitivity	Specificity	Positive predictive value	Negative predictive value	Accuracy
VIS-A	0.80 [0.65-0.90]	0.48 [0.29-0.68]	0.72 [0.63-0.79]	0.59 [0.42-0.74]	0.68 [0.56-0.79]
VIS-B	0.67 [0.43-0.85]	0.73 [0.50-0.89]	0.70 [0.53-0.83]	0.70 [0.54-0.82]	0.70 [0.54-0.83]
VIS-C	0.44 [0.14-0.79]	0.88 [0.70-0.98]	0.57 [0.27-0.83]	0.82 [0.72-0.89]	0.77 [0.60-0.90]

Table S3 – Pooled prediction performances of the STARHE-RISK classification model according to the LI-RADS visualisation score. Notes: 95% confidence in squared brackets.

	LI-RADS US-3 observations	Rate of detected lesions	Sensitivity	Specificity	Positive predicted value	Negative predictive value	Positive likelihood ratio	Negative likelihood ratio	Accuracy
**Reader 1 without deep learning assistance**	31	19/25 (76%)	0.76 (0.55-0.91)	0.76 (0.62-0.87)	0.61 (0.48-0.73)	0.86 (0.76-0.83)	3.17 (1.84-5.44)	0.32 (0.15-0.65)	0.76 (0.65-0.85)
**LI-RADS visualisation score**									
VIS-A (n = 51)	20	15/19 (79%)	0.79 (0.54-0.94)	0.84 (0.67-0.95)	0.75 (0.56-0.87)	0.87 (0.74-0.94)	5.05 (2.19-11.68)	0.25 (0.10-0.60)	0.82 (0.69-0.92)
VIS-B (n = 17)	8	3/4 (75%)	0.75 (0.19-0.99)	0.62 (0.32-0.86)	0.38 (0.20-0.59)	0.89 (0.58-0.98)	1.95 (0.80-4.75)	0.41 (0.07-2.34)	0.65 (0.38-0.86)
VIS-C (n = 7)	3	1/2 (50%)	0.50 (0.01-0.99)	0.60 (0.15-0.95)	0.33 (0.08-0.74)	0.75 (0.39-0.93)	1.25 (0.22-7.22)	0.83 (0.18-3.96)	0.57 (0.18-0.90)
**Nodule size**									
≤ 2.0 cm (n = 12)	20	8/12 (67%)	0.67 (0.35-0.90)	0.76 (0.62-0.87)	0.40 (0.26-0.56)	0.90 (0.81-0.96)	2.78 (1.47-5.24)	0.44 (0.19-0.99)	0.74 (0.62-0.84)
2.0-3.0 cm (n = 8)	18	6/8 (75%)	0.75 (0.35-0.97)	0.76 (0.62-0.87)	0.33 (0.21-0.49)	0.95 (0.85-0.98)	3.12 (1.66-5.90)	0.33 (0.10-1.10)	0.76 (0.63-0.86)
> 3.0 cm (n = 5)	17	5/5 (100%)	1.00 (0.48-1.00)	0.76 (0.61-0.87)	0.29 (0.20-0.41)	1.00 (0.91-1.00)	4.17 (2.54-6.82)	0.00 (NA)	0.78 (0.65-0.88)
**Nodule echogenicity**									
Hypoechoic (n = 7)	18	6/7 (86%)	0.86 (0.42-1.00)	0.76 (0.62-0.87)	0.33 (0.22-0.47)	0.97 (0.86-1.00)	3.57 (2.00-6.37)	0.19 (0.03-1.16)	0.77 (0.64-0.87)
Isoechoic (n = 8)	15	3/8 (38%)	0.38 (0.09-0.76)	0.76 (0.62-0.87)	0.20 (0.08-0.41)	0.88 (0.81-0.93)	1.56 (0.56-4.34)	0.82 (0.47-1.44)	0.71 (0.57-0.82)
Hyperechoic (n = 6)	18	6/6 (100%)	1.00 (0.54-1.00)	0.76 (0.62-0.87)	0.33 (0.23-0.45)	1.00 (0.91-1.00)	4.17 (2.54-6.82)	0.00 (NA)	0.79 (0.66-0.88)
Heterogeneous (n = 4)	16	4/4 (100%)	1.00 (0.40-1.00)	0.76 (0.62-0.87)	0.25 (0.17-0.35)	1.00 (0.91-1.00)	4.17 (2.54-6.82)	0.00 (NA)	0.78 (0.64-0.88)
**Reader 1 with deep learning assistance**	21	18/25 (72%)	0.72 (0.51-0.88)	0.94 (0.83-0.99)	0.86 (0.66-0.95)	0.87 (0.78-0.93)	12.00 (3.90-36.93)	0.30 (0.16-0.56)	0.87 (0.77-0.93)

**LI-RADS visualisation score**									
VIS-A (n = 51)	17	15/19 (79%)	0.79 (0.54-0.94)	0.94 (0.79-0.99)	0.88 (0.66-0.97)	0.88 (0.76-0.95)	12.6 (3.24-49.31)	0.22 (0.09-0.54)	0.88 (0.76-0.96)
VIS-B (n = 17)	3	2/4 (50%)	0.50 (0.07-0.93)	0.92 (0.64-1.00)	0.67 (0.19-0.94)	0.86 (0.69-0.94)	6.5 (0.78-54.31)	0.54 (0.20-1.46)	0.82 (0.57-0.96)
VIS-C (n = 7)	1	1/2 (50%)	0.50 (0.01-0.99)	1.00 (0.48-1.00)	1.00 (0.03-1.00)	0.83 (0.56-0.95)	NA	0.5 (0.13-2.00)	0.86 (0.42-1.00)
**Nodule size**									
≤ 2.0 cm (n = 12)	10	7/12 (58%)	0.58 (0.28-0.85)	0.94 (0.83-0.99)	0.70 (0.41-0.89)	0.90 (0.83-0.85)	9.72 (2.94-32.18)	0.44 (0.23-0.87)	0.87 (0.76-0.94)
2.0-3.0 cm (n = 8)	9	6/8 (75%)	0.75 (0.35-0.97)	0.94 (0.83-0.99)	0.67 (0.38-0.87)	0.86 (0.88-0.99)	12.5 (3.89-40.19)	0.27 (0.08-0.89)	0.91 (0.81-0.97)
> 3.0 cm (n = 5)	8	5/5 (100%)	1.00 (0.48-1.00)	0.94 (0.83-0.99)	0.63 (0.36-0.83)	1.00 (0.92-1.00)	16.67 (5.56-49.93)	0.00 (NA)	0.95 (0.85-0.99)
**Nodule echogenicity**									
Hypoechoic (n = 7)	9	6/7 (86%)	0.86 (0.42-1.00	0.94 (0.83-0.99)	0.67 (0.39-0.86)	0.98 (0.88-1.00)	14.29 (4.58-44.58)	0.15 (0.02-0.93)	0.93 (0.83-0.98)
Isoechoic (n = 8)	6	3/8 (38%)	0.38 (0.09-0.76)	0.94 (0.83-0.99)	0.50 (0.20-0.80)	0.90 (0.85-0.94)	6.25 (1.52-25.74)	0.66 (0.39-1.14)	0.86 (0.75-0.94)
Hyperechoic (n = 6)	9	6/6 (100%)	1.00 (0.54-1.00)	0.94 (0.83-0.99)	0.67 (0.40-0.86)	1.00 (0.92-1.00)	16.67 (5.56-49.93)	0.00 (NA)	0.95 (0.85-0.99)
Heterogeneous (n = 4)	6	3/4 (75%)	0.75 (0.19-0.99)	0.94 (0.83-0.99)	0.98 (0.90-1.00)	0.98 (0.90-1.00)	12.5 (3.64-42.96)	0.27 (0.05-1.45)	0.93 (0.82-0.98)
**Reader 2 without deep learning assistance**	23	19/25 (76%)	0.76 (0.55-0.91)	0.92 (0.81-0.98)	0.83 (0.64-0.93)	0.88 (0.79-0.94)	9.50 (3.62-24.95)	0.26 (0.13-0.53)	0.87 (0.77-0.93)
**LI-RADS visualization score**									
VIS-A (n = 31)	11	10/12 (83%)	0.83 (0.52-0.98)	0.95 (0.74-1.00)	0.91 (0.59-0.99)	0.90 (0.72-0.97)	15.83 (2.31-108.48)	0.18 (0.05-0.63)	0.90 (0.74-0.98)
VIS-B (n = 24)	9	7/8 (88%)	0.88 (0.47-1.00)	0.88 (0.62-0.98)	0.78 (0.48-0.93)	0.93 (0.69-0.99)	7.00 (1.87-26.27)	0.14 (0.02-0.90)	0.88 (0.68-0.97)
VIS-C (n = 20)	3	2/5 (40%)	0.40 (0.05-0.85)	0.93 (0.68-1.00)	0.67 (0.18-0.95)	0.82 (0.69-0.91)	6.00 (0.68-52.90)	0.64 (0.31-1.33)	0.80 (0.56-0.94)

**Nodule size**									
≤ 2.0 cm (n = 12)	11	7/12 (58%)	0.58 (0.28-0.85)	0.92 (0.81-0.98)	0.64 (0.38-0.83)	0.90 (0.82-0.95)	7.29 (2.54-20.93)	0.45 (0.23-0.89)	0.85 (0.74-0.93)
2.0-3.0 cm (n = 8)	11	7/8 (88%)	0.88 (0.47-1.00)	0.92 (0.81-0.98)	0.64 (0.40-0.82)	0.98 (0.88-1.00)	10.94 (4.12-29.02)	0.14 (0.02-0.85)	0.91 (0.81-0.97)
> 3.0 cm (n = 5)	9	5/5 (100%)	1.00 (0.48-1.00)	0.92 (0.81-0.98)	0.56 (0.33-0.76)	1.00 (0.92-1.00)	12.5 (4.88-32.00)	0.00 (NA)	0.93 (0.82-0.98)
**Nodule echogenicity**									
Hypoechoic (n = 7)	10	6/7 (86%)	0.86 (0.42-1.00)	0.92 (0.81-0.98)	0.60 (0.36-0.80)	0.98 (0.88-1.00)	10.71 (3.99-28.76)	0.13 (0.03-0.95)	0.91 (0.81-0.97)
Isoechoic (n = 8)	8	4/8 (50%)	0.50 (0.16-0.84)	0.92 (0.81-0.98)	0.50 (0.24-0.76)	0.92 (0.85-0.96)	6.25 (1.94-20.09)	0.54 (0.27-1.09)	0.86 (0.75-0.94)
Hyperechoic (n = 6)	10	6/6 (100%)	1.00 (0.54-1.00)	0.92 (0.81-0.98)	0.60 (0.67-0.79)	1.00 (0.92-1.00)	12.5 (4.88-32.00)	0 (NA)	0.93 (0.83-0.98)
Heterogeneous (n = 4)	7	3/4 (75%)	0.75 (0.19-0.99)	0.92 (0.81-0.98)	0.43 (0.20-0.69)	0.98 (0.89-1.00)	9.38 (3.13-28.08)	0.27 (0.05-1.49)	0.91 (0.80-0.97)
**Reader 2 with deep learning assistance**	27	19/25 (76%)	0.76 (0.55-0.91)	0.84 (0.71-0.93)	0.70 (0.55-0.82)	0.88 (0.78-0.93)	4.75 (2.43-9.30)	0.29 (0.14-0.58)	0.81 (0.71-0.89)
**LI-RADS visualisation score**									
VIS-A (n = 31)	12	9/12 (75%)	0.75 (0.43-0.95)	0.84 (0.60-0.97)	0.75 (0.50-0.90)	0.84 (0.66-0.94)	4.75 (1.6-14.11)	0.3 (0.11-0.81)	0.81 (0.63-0.93)
VIS-B (n = 24)	11	8/8 (100%)	1.00 (0.63-1.00)	0.81 (0.54-0.96)	0.73 (0.49-0.88)	1.00 (0.75-1.00)	5.33 (1.92-14.79)	0.00 (NA)	0.88 (0.68-0.97)
VIS-C (n = 20)	4	2/5 (40%)	0.40 (0.05-0.85)	0.67 (0.60-0.98)	0.50 (0.67-0.90)	0.81 (0.67-0.90)	3.00 (0.56-16.07)	0.69 (0.33-1.45)	0.75 (0.51-0.91)
**Nodule size**									
≤ 2.0 cm (n = 12)	16	8/12 (67%)	0.67 (0.35-0.90)	0.84 (0.71-0.93)	0.50 (0.32-0.68)	0.91 (0.82-0.96)	4.17 (1.97-8.83)	0.4 (0.18-0.89)	0.81 (0.69-0.90)
2.0-3.0 cm (n = 8)	14	6/8 (75%)	0.75 (0.35-0.97)	0.84 (0.71-0.93)	0.43 (0.36-0.61)	0.95 (0.86-0.99)	4.69 (2.21-9.93)	0.3 (0.09-0.99)	0.83 (0.71-0.91)
> 3.0 cm (n = 5)	13	5/5 (100%)	1.00 (0.48-1.00)	0.84 (0.71-0.93)	0.38 (0.25-0.54)	1.00 (0.92-1.00)	6.25 (3.31-11.80)	0 (NA)	0.85 (0.73-0.94)

**Nodule echogenicity**									
Hypoechoic (n = 7)	14	6/7 (86%)	0.86 (0.42-0.91)	0.84 (0.71-0.93)	0.43 (0.27-0.60)	0.98 (0.87-1.00)	5.36 (2.65-10.83)	0.17 (0.03-1.05)	0.84 (0.72-0.93)
Isoechoic (n = 8)	12	4/8 (50%)	0.50 (0.16-0.84)	0.84 (0.71-0.93)	0.33 (0.16-0.56)	0.91 (0.84-0.96)	3.12 (1.22-8.00)	0.6 (0.29-1.20)	0.79 (0.67-0.89)
Hyperechoic (n = 6)	14	6/6 (100%)	1.00 (0.54-1.00)	0.84 (0.71-0.93)	0.43 (0.28-0.59)	1.00 (0.92-1.00)	6.25 (3.31-11.80)	0.00 (NA)	0.86 (0.74-0.94)
Heterogeneous (n = 4)	11	3/4 (75%)	0.75 (0.19-0.99)	0.84 (0.71-0.93)	0.27 (0.174-0.47)	0.98 (0.88-1.00)	4.69 (2.00-10.97)	0.3 (0.05-1.63)	0.83 (0.71-0.92)
**Reader 3 without deep learning assistance**	24	19/25 (76%)	0.76 (0.55-0.91)	0.88 (0.76-0.95)	0.76 (0.59-0.87)	0.88 (0.78-0.94)	6.33 (2.90-13.85)	0.27 (0.13-0.55)	0.84 (0.74-0.91)
**LI-RADS visualisation score**									
VIS-A	13	10/13 (77%)	0.77 (0.46-0.95)	0.86 (0.64-0.97)	0.77 (0.53-0.91)	0.86 (0.69-0.94)	5.38 (1.81-16.00)	0.27 (0.10-0.74)	0.82 (0.65-0.93)
VIS-B	8	7/8 (88%)	0.88 (0.66-1.00)	0.93 (0.66-1.00)	0.88 (0.67-0.99)	0.93 (0.67-0.99)	12.25 (1.82-82.46)	0.13 (0.02-0.85)	0.91 (0.71-0.99)
VIS-C	4	2/4 (50%)	0.50 (0.07-0.93)	0.87 (0.60-0.98)	0.50 (0.17-0.83)	0.87 (0.71-0.95)	3.75 (0.74-18.95)	0.58 (0.21-1.57)	0.79 (0.54-0.94)
**Nodule size**									
≤ 2.0 cm (n = 12)	13	8/12 (67%)	0.67 (0.35-0.90)	0.90 (0.78-0.97)	0.62 (0.39-0.80)	0.92 (0.83-0.96)	6.67 (2.65-16.78)	0.37 (0.17-0.83)	0.85 (0.74-0.93)
2.0-3.0 cm (n = 8)	11	6/8 (75%)	0.75 (0.35-0.97)	0.90 (0.78-0.97)	0.55 (0.32-0.75)	0.96 (0.87-0.99)	7.50 (2.98-18.87)	0.28 (0.08-0.93)	0.88 (0.77-0.95)
> 3.0 cm (n = 5)	10	5/5 (100%)	1.00 (0.48-1.00)	0.90 (0.78-0.97)	0.50 (0.30-0.70)	1.00 (0.92-1.00)	10.00 (4.35-22.97)	0.00 (NA)	0.91 (0.80-0.97)
**Nodule echogenicity**									
Hypoechoic (n = 7)	12	6/7 (86%)	0.86 (0.42-1.00)	0.90 (0.78-0.97)	0.55 (0.33-0.74)	0.98 (0.88-1.00)	8.57 (3.54-20.77)	0.16 (0.03-0.98)	0.89 (0.78-0.96)
Isoechoic (n = 8)	10	4/8 (50%)	0.50 (0.16-0.84)	0.90 (0.78-0.97)	0.44 (0.21-0.70)	0.92 (0.85-0.96)	5.00 (1.69-14.76)	0.56 (0.28-1.12)	0.84 (0.76-0.93)
Hyperechoic (n = 6)	12	6/6 (100%)	1.00 (0.54-1.00)	0.90 (0.78-0.97)	0.55 (0.34-0.63)	1.00 (0.92-1.00)	10.00 (4.35-22.97)	0.00 (NA)	0.91 (0.80-0.97)

Heterogeneous (n = 4)	9	3/4 (75%)	0.75 (0.19-0.99)	0.90 (0.78-0.97)	0.38 (0.18-0.62)	0.98 (0.89-1.00)	7.50 (2.74-20.51)	0.28 (0.05-1.52)	0.89 (0.77-0.96)
**Reader 3 with deep learning assistance**	23	19/25 (76%)	0.76 (0.55-0.91)	0.92 (0.81-0.98)	0.83 (0.64-0.93)	0.88 (0.79-0.94)	9.50 (3.62-24.95)	0.26 (0.13-0.53)	0.87 (0.77-0.93)
**LI-RADS visualisation score**									
VIS-A	13	11/13 (85%)	0.85 (0.55-0.98)	0.90 (0.70-0.99)	0.85 (0.59-0.95)	0.90 (0.72-0.97)	8.88 (2.33-33.88)	0.17 (0.05-0.61)	0.88 (0.73-0.97)
VIS-B	7	6/8 (75%)	0.75 (0.35-0.97)	0.93 (0.66-1.00)	0.86 (0.47-0.98)	0.87 (0.66-0.96)	10.50 (1.52-72.38)	0.27 (0.08-0.90)	0.86 (0.65-0.97)
VIS-C	3	2/4 (50%)	0.50 (0.07-0.93)	0.93 (0.68-1.00)	0.67 (0.19-0.94)	0.88 (0.72-0.95)	7.50 (0.89-63.24)	0.54 (0.20-1.44)	0.84 (0.60-0.97)
**Nodule size**									
≤ 2.0 cm (n = 12)	12	8/12 (67%)	0.67 (0.35-0.90)	0.92 (0.81-0.98)	0.67 (0.42-0.85)	0.92 (0.84-0.96)	8.33 (3.00-23.15)	0.36 (0.16-0.81)	0.87 (0.76-0.94)
2.0-3.0 cm (n = 8)	10	6/8 (75%)	0.75 (0.35-0.97)	0.92 (0.81-0.98)	0.60 (0.35-0.81)	0.96 (0.87-0.99)	9.38 (3.38-26.04)	0.27 (0.08-0.90)	0.90 (0.79-0.96)
> 3.0 cm (n = 5)	9	5/5 (100%)	1.00 (0.48-1.00)	0.92 (0.81-0.98)	0.56 (0.33-0.76)	1.00 (0.92-1.00)	12.50 (4.88-32.00)	0.00 (NA)	0.93 (0.82-.098)
**Nodule echogenicity**									
Hypoechoic (n = 7)	10	6/7 (86%)	0.86 (0.42-1.00)	0.92 (0.81-0.98)	0.60 (0.36-0.80)	0.98 (0.88-1.00)	10.71 (3.99-28.76)	0.16 (0.03-0.95)	0.91 (0.81-0.97)
Isoechoic (n = 8)	8	4/8 (50%)	0.50 (0.16-0.84)	0.92 (0.81-0.98)	0.50 (0.24-0.76)	0.92 (0.85-0.96)	6.25 (1.94-20.09)	0.54 (0.27-1.09)	0.86 (0.75-0.94)
Hyperechoic (n = 6)	10	6/6 (100%)	1.00 (0.54-1.00)	0.92 (0.81-0.98)	0.60 (0.37-0.79)	1.00 (0.92-1.00)	12.50 (4.88-32.00)	0.00 (NA)	0.93 (0.83-0.98)
Heterogeneous (n = 4)	7	3/4 (75%)	0.75 (0.19-0.99)	0.92 (0.81-0.98)	0.43 (0.20-0.69)	0.98 (0.89-1.00)	9.38 (3.13-28.08)	0.27 (0.05-1.49)	0.91 (0.80-0.97)

Table S4 – Subgroup performances of the 3 radiologists without and with the assistance of the deep learning object detection model for detecting early-stage HCC (BCLC 0 or A)
