# Supplementary material for: Improving risk stratification and detection of early HCC using ultrasound-based deep learning models
Source: JHEP Rep. 2025 Jul 5;7(10):101510. doi: 10.1016/j.jhepr.2025.101510 (PMC12448012; doi:10.1016/j.jhepr.2025.101510)
Supplement: Multimedia component 4 [file mmc4.pdf]

Research article

JHEP|Reports

Improving risk stratification and detection of early HCC using ultrasound-based deep learning models

Authors

Jérémy Dana, Adrien Meyer, Anita Paisant, …, Benoit Gallix, Nicolas Padoy, Pierre Nahon

Correspondence

jeremy.dana@ext.ihu-strasbourg.eu (J. Dana).

Graphical abstract

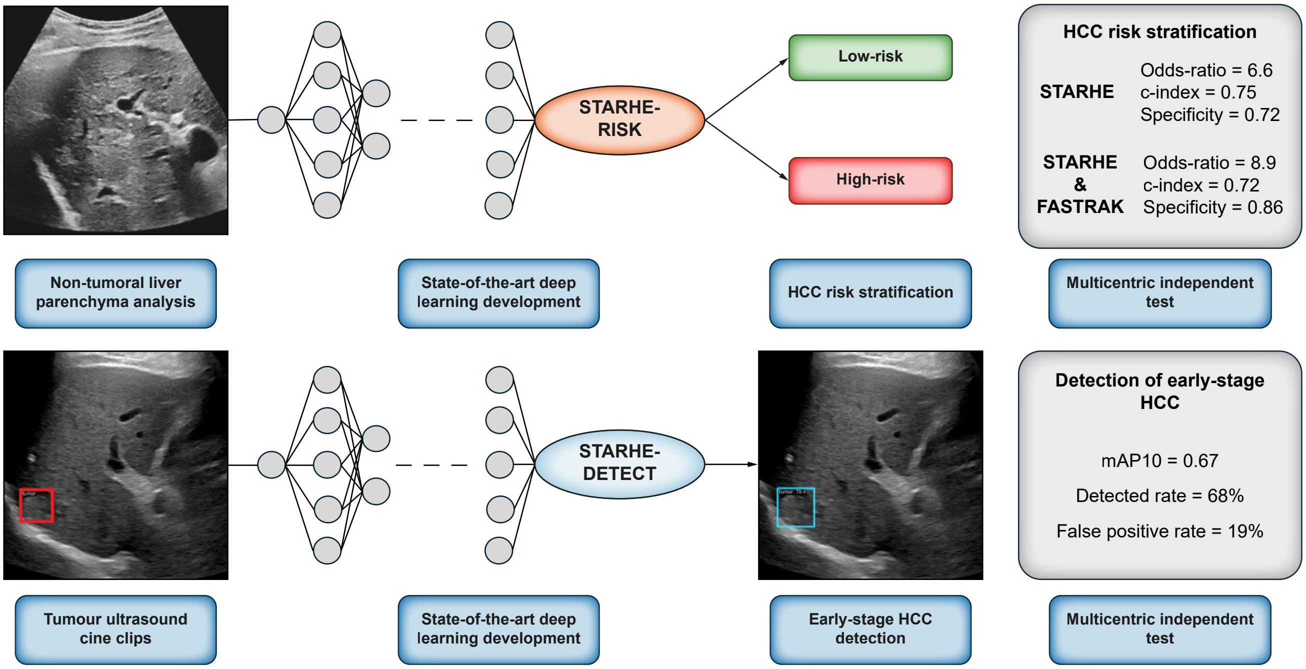

Highlights:

- HCC risk stratification can be achieved using liver ultrasound.
- Ultrasound, clinical, and blood scores can be combined for HCC risk stratification.
- STARHE-DETECT can become a valuable surveillance tool for early-stage HCC detection.
- This study paves the way for risk-based personalised surveillance programmes.

Impact and implications:

STARHE-RISK is a reliable ultrasound-based deep learning model for hepatocellular carcinoma (HCC) risk stratification in patients with compensated advanced chronic liver disease and can be associated with complementary scores integrating clinical and blood parameters. STARHE-DETECT could become a complementary tool to visual assessment for radiologists and sonographers in HCC surveillance. Both models are based on simple and easy-to-perform ultrasound cine clip acquisitions. This study paves the way for a risk-based personalised surveillance program that will not ultimately rely on a single test but rather on a combination of approaches mixing clinical, biological, and radiological data.

https://doi.org/10.1016/j.jhepr.2025.101510

© 2025 The Author(s). Published by Elsevier B.V. on behalf of European Association for the Study of the Liver (EASL). This is an open access article under the CC BY license (http://creativecommons.org/licenses/by/4.0/). JHEP Reports, 2025, 7, 1–13

Improving risk stratification and detection of early HCC using ultrasound-based deep learning models

Jérémy Dana[1,2,3,4,*], Adrien Meyer[5], Anita Paisant[6,7], Agnès Rode[8], Riccardo Sartoris[9,10], Olivier Séror[11], Christophe Cassinotto[12], Laurent Milot[13,14], Jules Grégory[9,15], Jules Cœur[8], Jérôme Lebigot[6], Valentina Schembri[16], François Villeret[17], Armelle-Natsuo Takeda[2], Maxime Ronot[9,10], Valérie Vilgrain[9,10], Thomas F. Baumert[1,2,18], Benoit Gallix[3,19,20,†], Nicolas Padoy[2,5,†], Pierre Nahon[21,22,23,†]

JHEP Reports **2025**. vol. 7 | 1–13

Check for updates

Background & Aims: Hepatocellular carcinoma (HCC) surveillance programs are suboptimal. We aimed to design an ultrasound-based deep learning model for HCC risk stratification (STARHE-RISK) and early-stage HCC detection (STARHE-DETECT) in patients with compensated advanced chronic liver disease (cACLD).

Methods: This prospective multicentric study included 403 adult patients with cACLD of all causes enrolled in a surveillance program for at least 6 months without prior history of HCC. STARHE-RISK was trained on ultrasound cine clips of the non-tumoral liver parenchyma using two classes: cases (n = 152 patients with early-stage HCC; 137/152 [82%] male; median age 63 years) and controls (n = 170 patients without HCC at inclusion and during a subsequent 1-year follow-up; 120/170 [71%] male; median age 69 years). STARHE-DETECT was trained on tumour ultrasound cine clips. The training/validation and testing sets were stratified according to potential confounders, and 50 patients who were balanced in both groups were allocated to the independent testing set based on sample size calculation. Statistical analysis included classification and detection metrics.

Results: STARHE-RISK achieved good prediction performances in the testing set with a 0.72 accuracy (95% CI 0.57–0.84) and an odds ratio of 6.6 (95% CI 1.9–22.7; p = 0.003). The combination of STARHE-RISK and the FASTRAK score, a multi-aetiology HCC risk stratification score, achieved a higher specificity (0.86 [95% CI 0.65–0.97]) and odds ratio (8.9 [95% CI 2.1–38.3; p = 0.004]) for predicting a patient at high risk of HCC development. STARHE-DETECT achieved a 0.67 mAP10, a 0.68 sensitivity (95% CI 0.47–0.85), and a 0.82 specificity (95% CI 0.69–0.91) for detecting early-stage HCC.

Conclusions: STARHE-RISK and STARHE-DETECT achieved robust performances for HCC risk stratification and early-stage HCC detection, respectively. They could become valuable surveillance tools and pave the way for a risk-based personalised surveillance program.

Clinical Trials Registration: The study is registered at ClinicalTrials.gov (NCT04802954).

© 2025 The Author(s). Published by Elsevier B.V. on behalf of European Association for the Study of the Liver (EASL). This is an open access article under the CC BY license (http://creativecommons.org/licenses/by/4.0/).

Introduction

Hepatocellular carcinoma (HCC), a life-threatening condition, arises in more than 80% of cases in cirrhosis in the West. Over the past decades, the incidence rate of liver cancers has been increasing, and the severity of this challenge is amplified by projections that anticipate a 55% increase in new cases of liver cancer by 2040, which would result in 1.3 million deaths worldwide – a 56% increase compared with 2020.[1]

In this context, healthcare systems have included patients with compensated advanced chronic liver disease in surveillance programs with biannual ultrasound. However, although abdominal ultrasound is an inexpensive and radiation-free examination, it has significant shortcomings in sensitivity and interobserver reproducibility, particularly for detecting early-stage HCC with a reported sensitivity of 47%[2] and even lower for HCC <2 cm, where sensitivity drops to 22%.[3] Indeed, in routine practice, only about 20% of patients diagnosed with HCC receive a first-line curative-intent treatment.[4] Thus, surveillance by magnetic resonance imaging (MRI), especially with abbreviated protocols, has been reported to improve surveillance as it significantly outperforms ultrasound with a detection rate of five times that of ultrasound for very early-stage (<2 cm) HCC.[5] The reported sensitivity and specificity ranged from 84.6 to 96.0% and from 81.6 to 100%.[6–12] However, although the diagnostic performance of abbreviated MRI is superior to that of ultrasound, MRI is an expensive examination with limited access. This is why there is an urgent unmet clinical need to identify a subpopulation at very high risk of HCC development while also improving the performance of ultrasound for early-stage HCC surveillance.

* Corresponding author. Address: IHU Strasbourg - Institut de chirurgie guidée par l'image, 1 Place de l'Hôpital, 67000 Strasbourg, France. Tel.: +33 3 90 41 36 00; Fax: +33 3 90 41 36 99.
E-mail address: jeremy.dana@ext.ihu-strasbourg.eu (J. Dana).
† These authors share last authorship.
https://doi.org/10.1016/j.jhepr.2025.101510

Indeed, surveillance programs rely on the cost-effectiveness ratio at the population's collective level, which is determined by the incidence of HCC, the cost of the surveillance tools and its benefits, including the percentage of patients receiving a first-line curative-intent treatment and the overall survival in at-risk patients. Recent analyses of prospective European cohorts, including a model-based evaluation of very early-stage HCC detection, confirmed that MRI surveillance is cost-effective for a baseline yearly incidence of 3% in patients with cirrhosis without active viral replication, which was similar to that of Asian populations.[5,13] Therefore, surveillance with abbreviated MRI can only be considered for a subpopulation with a very high risk of HCC development, which would be selected from the population currently undergoing standard ultrasound surveillance. Identifying this subset of high-risk patients is crucial as this strategy would detect five times more very early-stage HCC than ultrasound, with an incremental cost-effective ratio below 30,000€/life-years gained.[14] Refining and personalising costly HCC surveillance programs based on the individual risk of HCC is a timely challenge to provide better care and allocate limited medical resources fairly.

Numerous algorithms have been developed, either aetiology-specific[15–17] or multi-aetiology,[5] incorporating clinical parameters (*e.g.* age, sex, BMI, or diabetes) and biological parameters (*e.g.* gamma-glutamyl transferase [GGT], aspartate aminotransferase [AST]/alanine aminotransferase [ALT], platelets, or albumin),[16,18,19] serum proteins,[20–22] or single nucleotide polymorphisms.[23] These models demonstrated good discriminative performances. For instance, Nahon *et al.*[5] developed a multi-aetiology score (FASTRAK) dedicated to European patients with cirrhosis and no viral replication based on age, sex, platelet count, total bilirubin, GGT, and serum α-foetoprotein (AFP) that achieved a Harrell's c-index up to 0.76 to identify patients with an annual risk of HCC over 3% after 3 years of follow-up.[5] However, these models do not take into consideration the structural analysis of the liver parenchyma, which reflects the pathophysiological mechanisms responsible for hepatocarcinogenesis. In the 1990s, ultrasound studies examined the incidence of HCC according to the liver echostructure.[24–26] Results showed that a nodular heterogeneous echostructure resulted in an adjusted rate ratio estimate of up to 20.

Therefore, we hypothesised that non-tumoral liver parenchyma of patients eligible for HCC surveillance is rich in structural information reflecting the severity of liver disease and the risk of HCC development. We also hypothesised that the performance of ultrasound in early-stage HCC surveillance could be improved with the assistance of a deep learning object detection model. Indeed, object detection deep learning models have become increasingly popular in assisting radiologists in surveillance programs such as mammography breast cancer screening.[27,28]

The primary objective was (1) to design a deep learning model for HCC risk stratification based on ultrasound cine clips of the non-tumoral liver parenchyma in patients with compensated advanced chronic liver disease (cACLD) to identify a subpopulation at very high risk of HCC development and (2) to develop an object detection model for early-stage HCC on surveillance ultrasound cine clips in patients with cACLD. The secondary objective was to assess the impact of the object detection model on radiologists' interpretation of surveillance ultrasound cine clips.

Materials and methods

Ethics

This prospective project was approved by the Research Ethics Board (Comité de protection des personnes Sud-Est VI 21.03054.001701-MS03; ClinicalTrial NCT04802954) and followed the ethical principles of the Declaration of Helsinki. All patients provided written informed consent.

Study design

This prospective multicentric study was conducted in six academic tertiary hospitals (CHU Angers, Angers, France; Hôpital de la Croix Rousse, Hospices Civiles de Lyon, Lyon, France; Hôpital Beaujon, Assistance Publique - Hôpitaux de Paris, Clichy, France; Hôpital Avicenne, Assistance Publique - Hôpitaux de Paris, Bobigny, France; Hôpital Saint Eloi, CHU Montpellier, Montpellier, France; Hôpital Edouard Herriot, Hospices Civiles de Lyon, Lyon, France). The study prospectively consecutively included patients over 18 years of age, enrolled in a surveillance program for at least 6 months, defined by Child–Pugh A or B histologically proven F3/F4 liver or cirrhosis unequivocally suggested by non-invasive tests of non-viral or controlled/healed B/C viral cause (HBV PCR negative under antiviral B treatment for more than 12 weeks/HCV PCR negative at least 12 weeks after stopping antiviral C treatment), referred by hepatologists for ultrasound surveillance, without a history of treated HCC. Patients without recorded imaging data (ultrasound cine clips) or who were lost to follow-up were excluded.

Primary endpoint

The primary endpoint of the study was (1) the prediction (classification) performances of the deep learning model for HCC risk stratification and (2) the performance of the deep learning model for the detection of very early (*i.e.* Barcelona-Clinic Liver Cancer [BCLC] stage 0, *i.e.* single tumour <2 cm) or early-stage HCC (*i.e.* BCLC A, *i.e.* one nodule of any size or ≤3 nodules, each <3 cm in diameter).[29]

Reference standard

A composite reference standard was used, including pathology (biopsy, surgical resection, and explant), radiology (LR-5 category per the Liver Imaging Reporting and Data System [LI-RADS] v2018 on dedicated computed tomography [CT] or MRI of the liver), and follow-up (1-year follow-up by ultrasound or dedicated liver CT or MRI if clinically warranted).

Two groups of patients were constituted:

- *HCC group:* Patients with early-stage HCC (BCLC 0 or A HCC) as per the reference diagnostic standards detected during surveillance at the time of inclusion and secondarily confirmed by the local multidisciplinary tumour board. The ultrasound examination at the time of study inclusion was performed after dedicated liver CT or MRI imaging.
- *Control group:* Patients without HCC at the time of inclusion. A subsequent 1-year interval ultrasound, or dedicated liver CT, or MRI if clinically warranted, was performed to confirm the

absence of new lesions in the year following the inclusion. The proportion of new HCC was expected not to exceed 3-5%. In the case of new HCC, these patients were reassigned to the cases group.

Collected data

All clinical, biological, imaging, and pathologic data were collected at inclusion.

- *Clinical:* demographics (age and sex), BMI, liver disease history (aetiology, viral hepatitis status, alcohol consumption), medical history (diabetes, HIV co-infection).
- *Biology:* liver disease scores (FASTRAK – a multi-aetiology score based on age, sex, platelet count, total bilirubin, GGT, and AFP that achieved a Harrell's c-index up to 0.76 to identify patients with an annual HCC risk of >3% after 3 years of follow-up – model for end-stage liver disease, Child–Pugh), tumour markers (AFP), liver function tests (bilirubin, AST, ALT, GGT), haemostasis (platelets, international normalised ratio [INR], prothrombin time), albumin.
- *Imaging:* ultrasound cine clips (non-tumoral liver parenchyma and HCC using B-mode ultrasound).
- *Pathology:* pathology report of the non-tumoral liver parenchyma and HCC if available.

Ultrasound examination

Ultrasound examinations were performed using two scanners from two manufacturers: Aplio (Canon Medical Systems, Otawara, Japan) and Aixplorer/MACH 30 (Supersonic Imagine, Aix-en-Provence, France; former Hologic, Marlborough, MA, USA). Conventional liver ultrasound was performed using B-mode and colour Doppler. Data acquisition was standardised according to a mandatory protocol implemented in each ultrasound scanner using a low-frequency abdominal convex transducer (C6-1X for Hologic SuperSonic Image and i8Cx1 for Canon Medical Systems): one 10-s B-mode cine clip acquired in free breathing and recorded in an intercostal section of the non-tumoral right liver without passing through the HCC (labelled B-mode non-tumoral liver) and one 10-s B-mode cine clip acquired in free breathing of the liver passing through the HCC for each visible HCC (labelled B-mode HCC). A default abdominal preset was used. Depth was initially set at 12 cm with a focal at 7.68 cm on Canon Medical Systems ultrasound and 7–8 cm on Hologic/SuperSonic Imagine ultrasound, but ultimately left to the operator's discretion.

2D ShearWave elastography was performed with low-frequency abdominal convex probes (C6-1X for Hologic SuperSonic Image and i8Cx1 for Canon Medical Systems). Liver stiffness measurements were acquired in an intercostal section in the right liver lobe using a fixed-size stiffness colour mapping. Three to five measurements were performed in the right liver according to the reference quality standards.[30]

Deep learning methodology

- *Database:* The training/validation and test (50 patients) sets were stratified according to potential confounders: aetiology of liver disease, FASTRAK score (a multi-aetiology risk stratification score based on age, sex, platelet count, total bilirubin, GGT, and AFP; binary cut-off of 9 points[31]), ultrasound manufacturer, HCC size (binary cut-off of 2 cm) and echogenicity (isoechoic to the background liver or not). To compensate for the imbalance between the two groups, we applied oversampling with data augmentation and weighted loss to penalise model errors for data from the minority group.
- *Labelling of the database:* Ultrasound videos were annotated and labelled by a radiologist subspecialised in liver imaging (XX – anonymised – with 3 years of experience, who did not participate in the cine clips review) using the MOSaiC Annotation Platform, a cloud-based collaborative video annotation platform.[32] HCCs were annotated with bounding boxes using a standardised annotation pipeline with keyframe interpolation.
- *Pre-processing of ultrasound images:* Ultrasound images were embedded in video layouts, informed by factors such as ultrasound machine brand and display settings. To standardise these images and minimise bias, we developed an automated method to extract the region of interest from the layout. Our method detected pixels with minimal intensity changes across video timestamps, classifying them as background. This classification allowed the creation of a binary mask. We then refined this mask using morphological operations to remove artefacts and cropped it around the ultrasound region of interest.
- *HCC risk stratification based on the non-tumoral liver parenchyma (STARHE-RISK model):* Based on a short video clip of non-tumoral liver parenchyma, we framed the task as a video classification challenge to identify patients at high risk of developing HCC. The video was divided into 10 clips with 16-frame subclips sampled from each. We designed a voting system between the model predictions for each subclip to determine the final classification of the video. Our implementation was based on the MMAction2 library. The training/validation set was split into five folds, and we performed cross-validation for model selection and hyperparameters tuning on the latter set (Table S1).[33] We selected three state-of-the-art algorithms with different sets of hyperparameters: MViT,[34] C3D,[35] and I3D.[36] Each model was pretrained on the Kinetics-400 dataset[37] to facilitate transfer learning. First, for each cross-validation session, all three models were trained with different sets of parameters, and their performances were measured on the validation folds. We selected the model and hyperparameter set with the best average performance across all folds as the final model. This final model was retrained using all the training/validation data. Grad-CAM++ explainability maps have been computed.[38]
- *Early-stage HCC detection (STARHE-DETECT model):* We aimed to develop an object detection model to detect and localise HCC as the video was played, highlighting the HCC with a bounding box. We selected three state-of-the-art models: Faster-RCNN, DINO-DETR, and RTMDet. Each model was pretrained on the COCO dataset to facilitate transfer learning. Our implementation was based on the MMDetection library. We used a stratified validation set of 15 patients to select the model architecture and tune the hyperparameters (Table S2).
- *Independent testing and sample size calculation:* To ensure the robustness and generalizability of the deep learning model, we planned to test it in an independent dataset of 50 patients. Approaching this question from a statistical perspective, we included 50 patients in the independent testing dataset (significance level α of 5%, statistical power of 80%, control-to-

case ratio of 1:1, annual incidence of HCC of 3% in the control group and a relative risk of 11 between both groups). This estimate is based on previous studies showing that a macronodular heterogeneous echostructure on ultrasound is associated with an adjusted relative risk of up to 20.[24–26] Therefore, considering the requirements of deep learning model developments, we intended to include 400 patients (200 patients in the cases and control groups) to allow an approximate balance of 80–20% between the training/validation and testing sets and to compensate with excluded patients (expected rate of 10%). We also intended to simulate a test dataset to represent current surveillance practice with homogeneous and heterogeneous livers, and focal liver lesions. Therefore, we tested the STARHE-DETECT model on the B-mode HCC and B-mode non-tumoral liver ultrasound cine clips, resulting in a total of 75 ultrasound cine clips: 25 B-mode HCC cine clips, 25 B-mode non-tumoral liver cine clips from the control group hypothesised to represent more homogeneous liver, and 25 B-mode non-tumoral liver cine clips from the HCC group, hypothesised to represent more heterogeneous liver. In contrast, the STARHE-RISK model was only tested on the 50 B-mode non-tumoral liver cine clips.

Comparison with radiologists' assessment

The 75 ultrasound cine clips of the test dataset were independently reviewed by three board-certified fellowship-trained abdominal radiologists (XX, XX, and XX, with 6, 8, and 35 years of experience – anonymised). Each radiologist blindly graded the visualisation LI-RADS score (VIS-A: no or minimal limitations; VIS-B: moderate limitations; VIS-C: severe limitations – LI-RADS® US Surveillance v2024 Core[39]) and the echotexture of the non-tumoral liver parenchyma (normal – 0 point, increased homogeneous echogenicity – 1 point, coarse – 2 points, nodular – 3 points, macronodular – 4 points). They also assigned a detection category (US-1: negative; US-2: subthreshold with a focal observation <10 mm; US-3: positive with a focal observation ≥10 mm). The reviews were conducted first without and then during a separate session with the assistance of the STARHE-DETECT model, using the MOSaiC Annotation Platform.[32] The predictions of the detection model were displayed using bounding boxes.

Statistical analysis

Performance metrics of the STARHE-RISK model and echotexture classes assessed by the radiologists were computed for sensitivity, specificity, accuracy, positive and negative predictive values, positive and negative likelihood ratios, area under the receiver operating characteristics curve, c-index, and odds ratio. Calibration curve was computed and calibration was assessed using the Hosmer-Lemeshow goodness-of-fit test. Performance metrics were also computed for the combination of the STARHE-RISK and the FASTRAK score. The combined score was considered positive when both independent scores were positive in a same patient.

Performance metrics of the STARHE-DETECT model were computed using mean average precision (mAP; area under the precision-recall curve) with a predefined intersection over union of 10, 50, and 75%. Confusion matrices were computed for each patient at different confidence levels for an intersection over the union of 10% to assess the rate of true positive and false positive. Additional performance metrics included sensitivity, specificity, accuracy, positive and negative predictive values, and positive and negative likelihood ratios. A 10% threshold for the intersection over the union between the predicted box and the annotated box was chosen for further analysis and experiments because of the surveillance strategy paradigm, where detecting a lesion (surveillance sensitivity) is more important than accurately delineating it. Radiologists' performances with and without the detection model were compared with a McNemar's test.

The inter-reader agreement was calculated using Fleiss kappa.[40] The agreement was interpreted according to the kappa value as follows: <0 (poor); 0–0.2 (slight); 0.21–0.40 (fair); 0.41–0.60 (moderate); 0.61–0.80 (substantial); 0.81–1.00 (almost perfect).[41] Ordinal non-normally distributed data were compared with the Mann–Whitney test. All statistical analysis was performed using MedCalc Statistical Software version 23.0.5 (MedCalc Software bv, Ostend, Belgium; https://www.medcalc.org) and IBM Corp. Released 2023. IBM SPSS Statistics for Windows, Version 29.0.2.0 Armonk, NY, USA.

Results

Study group

This study enrolled 403 patients between September 2021 and December 2023, including 152 patients in the cases group (Fig. 1). Eighty-one patients were excluded: 44 did not match

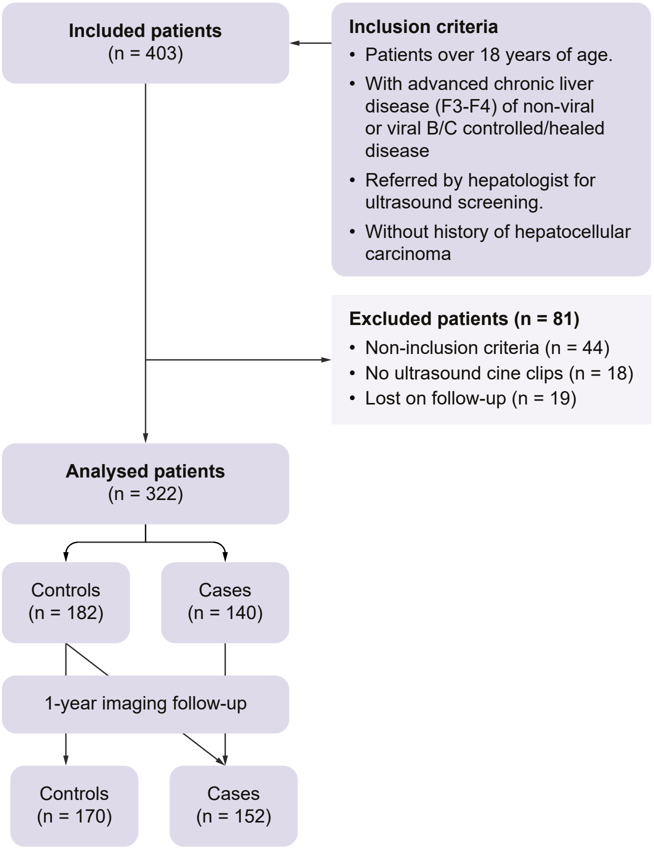

Fig. 1. Flow chart of the study.

Table 1. Demographic description of the population.

	Training/validation		Testing		Total	
	**Controls (n = 145)**	**Cases (n = 127)**	**Controls (n = 25)**	**Cases (n = 25)**	**Controls (n = 170)**	**Cases (n = 152)**
Centres						
1	28 (19%)	42 (33%)	3 (12%)	6 (24%)	31 (18%)	48 (32%)
2	31 (21%)	17 (52%)	5 (20%)	6 (24%)	36 (21%)	23 (15%)
3	27 (19%)	21 (17%)	2 (8%)	3 (12%)	29 (17%)	24 (16%)
4	1 (1%)	36 (28%)	0	7 (28%)	1 (1%)	43 (28%)
5	50 (34%)	8 (6%)	10 (40%)	2 (8%)	60 (35%)	10 (7%)
6	8 (6%)	3 (2%)	5 (20%)	1 (4%)	13 (8%)	4 (3%)
Ultrasound manufacturer						
Canon	51 (35%)	93 (73%)	9 (36%)	18 (72%)	60 (35%)	111 (73%)
Supersonic Imagine	94 (65%)	34 (27%)	16 (64%)	7 (28%)	110 (65%)	41 (27%)
Age (years)	63 [57-68]	69 [63-75]	61 [56-70]	68 [61-74]	63 [56-69]	69 [62-75]
Sex						
Male	102 (70%)	113 (89%)	18 (72%)	24 (96%)	120 (71%)	137 (81%)
Female	43 (30%)	14 (11%)	7 (28%)	1 (4%)	50 (29%)	15 (19%)
**Chronic liver disease**						
Aetiology of liver disease						
ALD	63 (44%)	47 (37%)	11 (44%)	8 (32%)	74 (44%)	55 (36%)
MASLD	27 (19%)	15 (16%)	5 (20%)	6 (24%)	32 (19%)	21 (14%)
MetALD	23 (16%)	31 (12%)	3 (12%)	3 (12%)	26 (15%)	34 (22%)
HBV	6 (4%)	3 (2%)	1 (4%)	1 (4%)	7 (4%)	4 (3%)
HCV	15 (10%)	14 (11%)	3 (12%)	2 (8%)	18 (11%)	16 (11%)
ALD + HBV	0 (0%)	1 (1%)	0 (0%)	0 (0%)	0 (0%)	1 (1%)
ALD + HCV	3 (2%)	4 (3%)	1 (4%)	5 (20%)	4 (2%)	9 (6%)
MASLD + HBV	1 (1%)	1 (1%)	0 (0%)	0 (0%)	1 (1%)	1 (1%)
MASLD + HCV	1 (1%)	0 (0%)	0 (0%)	0 (0%)	1 (1%)	0 (1%)
HBV + HCV	0 (0%)	1 (1%)	0 (0%)	0 (0%)	0 (0%)	1 (1%)
Other	6 (4%)	10 (8%)	1 (4%)	0 (0%)	7 (4%)	10 (6%)
FASTRAK score	7 [4-11]	10 [9-13]	8 [5-11]	11 [9-13]	7 [5-11]	10 [9-13]
Child-Pugh						
A5	88 (59%)	70 (55%)	15 (60%)	13 (52%)	103 (61%)	83 (55%)
A6	24 (19%)	27 (21%)	3 (12%)	3 (12%)	27 (16%)	30 (20%)
B7	12 (8%)	10 (8%)	1 (4%)	2 (8%)	13 (8%)	12 (8%)
B8	3 (21%)	1 (1%)	0 (0%)	2 (8%)	3 (2%)	3 (2%)
B9	0 (0%)	2 (2%)	0 (0%)	0 (0%)	0 (0%)	2 (1%)
Missing data	18 (12%)	17 (13%)	6 (24%)	5 (20%)	24 (14%)	22 (14%)
Type 2 diabetes	61 (42%)	52 (41%)	7 (28%)	10 (40%)	68 (40%)	62 (41%)
BMI ≥ 25	104 (72%)	87 (69%)	18 (72%)	19 (76%)	122 (72%)	106 (70%)
**Biology**						
Alpha-foetoprotein (ng/ml)	4.1 [2.6-5.2]	5.7 [3.0-10.5]	3.2 [2.4-5.5]	5.7 [3.6-7.0]	4.1 [2.6-5.2]	5.7 [3.0-10.5]
GGT (IU/L)	87 [39-161]	131 [64-270]	83 [36-124]	118 [58-267]	87 [39-161]	131 [64-270]
Total Bilirubin (μmol/L)	14 [9-19]	15 [9-23]	14 [11-24]	14 [10-28]	14 [9-19]	15 [9-23]
Platelet (G/L)	158 [107-200]	126 [95-172]	158 [99-190]	115 [82-201]	158 [107-201]	126 [95-172]
INR	1.2 [1.1-1.3]	1.1 [1.1-1.3]	1.1 [1.0-1.2]	1.1 [1.0-1.2]	1.2 [1.1-1.3]	1.1 [1.1-1.3]
Albumin (g/L)	41 [36-44]	40 [36-43]	42 [40-44]	39 [32-43]	41 [36-44]	40 [36-42]
**Shear wave elastography (kPa)**						
	14.4 [10.1-21.9]	12.8 [9.9-18.9]	13.1 [10.3-20.9]	11.3 [9.0-16.0]	14.1 [10.1-21.9]	12.5 [9.8-19.0]

(*continued on next page*)

Table 1. (*continued*)

	Training/validation		Testing		Total	
	Controls (n = 145)	Cases (n = 127)	Controls (n = 25)	Cases (n = 25)	Controls (n = 170)	Cases (n = 152)
**Hepatocellular carcinoma (at inclusion)**						
Number of nodules						
1	NA	87 (69%)	NA	18 (72%)	NA	105 (75%)
2		22 (17%)		7 (28%)		29 (21%)
3		6 (5%)		0		6 (4%)
Largest nodule size (mm)	NA	25 [20-31]	NA	22 [20-30]	NA	25 [20-31]
Nodule echogenicity	NA		NA		NA	
Homogeneous						
Hypoechoic		33 (37%)		8 (28%)		41 (36%)
Isoechoic		24 (27%)		8 (28%)		32 (28%)
Hyperechoic		19 (21%)		5 (20%)		24 (21%)
Heterogeneous						
Iso and hypoechoic		6 (7%)		2 (8%)		8 (7%)
Iso and hypoechoic		4 (4%)		1 (4%)		5 (4%)
Hypo and hyperechoic		4 (4%)		1 (4%)		5 (4%)
BCLC stage						
0	NA	31 (24%)	NA	10 (40%)	NA	41 (29%)
A		84 (76%)		15 (60%)		99 (71%)
**Ultrasound cine clips (B mode)**						
Data						
Non-tumor liver parenchyma	145 (100%)	122 (96%)	25 (100%)	25 (100%)	170 (100%)	147 (97%)
Hepatocellular carcinoma	NA	122 (96%)	NA	25 (100%)	NA	147 (97%)

AI, autoimmune; ALD, alcohol-related liver disease; BCLC, Barcelona-Clinic liver cancer; GGT, gamma-glutamyl transferase; HBV, Hepatitis B virus; HCV, Hepatitis C virus; INR, international normalised ratio; MASLD, metabolic dysfunction-associated steatotic liver disease; MetALD, MASLD, and ALD; Other (autoimmune, primary biliary cholangitis, granulomatosis, haemochromatosis, Wilson disease, iatrogenic).

the inclusion criteria, 19 were lost to follow-up, and 18 had no ultrasound cine clips recorded. In the training/validation dataset, 272 patients were analysed, including 145 patients in the control group and 127 in the cases group. In the independent testing dataset, 50 patients were analysed, including 25 patients in both groups (total of 75 cine clips).

Table 1 summarises the demographic description of the dataset.

HCC risk stratification – STARHE-RISK model

In the independent testing set, the trained C3D classification model achieved good diagnostic performances with an accuracy of 0.72 (95% CI 0.57–0.84) and a Harrell's c-index of 0.75 (95% CI 0.63–0.88) (Table 2). The model achieved an odds ratio of 6.6 to predict patients at high risk of HCC development (95% CI 1.9–22.7; p = 0.003). However, the C3D model achieved good calibration (p = 0.41 using the Hosmer-Lemeshow test – Fig. 2). In addition, poor quality of the ultrasound cine clip (LI-RADS VIS-C) was associated with decreased sensitivity of the model but increased specificity (Table S3). Fig. 3 illustrates two examples of a patient predicted at low risk and a patient predicted at high risk. An example of Grad-CAM++ explainability map is presented in Fig. S1, highlighting the importance of the liver in the prediction.

The FASTRAK score achieved slightly poorer diagnostic performances with an accuracy of 0.65 (95% CI 0.50–0.79), a Harrell's c-index of 0.65 (95% CI 0.53–0.77), and an odds ratio of 3.8 (95% CI 1.0–13.8; p = 0.04) for predicting a patient at high risk of developing an HCC. The combination of STARHE-RISK and the FASTRAK score (both positive in a same patient) achieved a higher specificity (0.86 [95% CI 0.65–0.97]), positive likelihood ratio of 4.28 (95% CI 1.42–12.91), negative likelihood ratio of 0.48 (95% CI 0.29–0.80) and odds ratio (8.9 [95% CI 2.1–38.3; p = 0.004]) for predicting a patient at high risk of developing HCC, outperforming both STARHE-RISK and the FASTRAK score when considered separately.

Detection of early-stage (BCLC 0 and A) HCC – STARHE-DETECT model

RTMDet achieved robust performances in the test set with a mAP10 of 0.67 and a mAP50 of 0.50 (Fig. 2). Fig. S2 illustrates the detection and false positive rates on the 25 B-mode HCC ultrasound cine clips of the patients in the HCC group across different confidence levels in the predicted deep learning bounding box.

With a 10% threshold for the intersection over the union between the predicted box and annotated box and a confidence level of 70%, the deep learning model achieved good to excellent performances with a sensitivity of 0.68 (95% CI 0.47–0.85), a specificity of 0.82 (95% CI 0.69–0.91), a positive likelihood ratio of 3.78 (95% CI 1.97–7.24), a negative likelihood ratio of 0.39 (95% CI 0.22–0.70) and an accuracy of 0.77 (95% CI 0.66–0.86) (Table 3).

When stratified on nodule size, the performances were highest for nodules >3.0 cm with a sensitivity of 0.80 but remained good for nodules ≤2.0 cm with a sensitivity of 0.67 (Fig. 4). However, the performances were highest for homogeneous hypoechoic and hyperechoic nodules (sensitivities of 0.75 and 1.00, respectively) but decreased for homogeneous isoechoic and heterogeneous nodules (sensitivity of 0.50 for

Table 2. Prediction performances of the STARHE-RISK classification model, FASTRAK score, and radiologists in the testing set. 95% CIs are shown in squared brackets.

	Sensitivity	Specificity	Positive predictive value	Negative predictive value	Accuracy	AUC	Harrell's c-index	Odds ratio
STARHE-RISK	0.72 [0.51–0.88]	0.72 [0.51–0.88]	0.72 [0.57–0.83]	0.72 [0.57–0.83]	0.72 [0.57–0.84]	0.71 [0.58–0.85]	0.75 [0.63–0.88]	6.6 [1.9–22.7] p = 0.003
FASTRAK score	0.79 [0.58–0.93]	0.50 [0.28–0.72]	0.63 [0.52–0.73]	0.69 [0.48–0.84]	0.65 [0.50–0.79]	0.65 [0.49–0.78]	0.65 [0.53–0.77]	3.8 [1.0–13.8] p = 0.04
STARHE-RISK + FASTRAK	0.58 [0.37–0.78]	0.86 [0.65–0.97]	0.82 [0.61–0.93]	0.66 [0.54–0.76]	0.72 [0.57–0.84]	0.72 [0.57–0.87]	0.72 [0.61–0.83]	8.9 [2.1–38.3] p = 0.004
**Echotexture**								
Coarse or nodular	0.60 [0.39–0.79]	0.60 [0.39–0.79]	0.60 [0.46–0.73]	0.60 [0.46–0.73]	0.60 [0.45–0.74]	0.60 [0.45–0.74]	0.60 [0.47–0.73]	2.3 [0.73–7.0] p = 0.16
Nodular	0.24 [0.09–0.45]	0.84 [0.64–0.95]	0.60 [0.32–0.82]	0.53 [0.39–0.68]	0.54 [0.39–0.68]	0.54 [0.39–0.68]	0.54 [0.44–0.64]	1.7 [0.41–6.8] p = 0.48

95% CIs are shown in squared brackets.

both). As expected, the performances were highest in VIS-A cine clips with a pooled sensitivity of 0.73 and lowest in VIS-C cine clips with a pooled sensitivity of 0.45.

Comparison with radiologists' assessment

The LI-RADS visualisation score was scored VIS-A in 31–51/75 (41–68%) cine clips, VIS-B in 17–24/75 (23–32%) cine clips, and VIS-C in 7–20/75 (9–27%) cine clips (range for the three readers). Inter-reader agreement was 0.36 (95% CI 0.27–0.46).

Regarding the echotexture assessment, the mean score between both readers was not statistically significantly different (p = 0.16) with a score of 1.6 in control patients and 2.0 in cases patients. The inter-reader agreement was 0.22 (95% CI 0.14–0.31). When the echotexture was scored coarse or micro/macronodular, the performances were moderate with a Harrell's c-index of 0.60 (95% CI 0.47–0.73) and an odds ratio of 2.3 (95% CI 0.73–7.0; p = 0.16) (Table 2).

Regarding the detection of early-stage HCC, the three readers achieved robust performances without and with the assistance of the STARHE-DETECT model with a pooled sensitivity of 0.76 (95% CI 0.65–0.85) and 0.75 (95% CI 0.63–0.84), a pooled specificity of 0.86 (95% CI 0.79–0.91) and 0.90 (95% CI 0.84–0.94), and a pooled positive likelihood ratio of 5.43 (95% CI 3.58–8.23) and 7.47 (95% CI 4.54–12.28), respectively (Table 4). There were no statistically significant differences for the three readers (p = 0.11 for reader 1, p = 0.57 for reader 2, and p = 0.81 for reader 3). For two out of the three readers, the number of positive observations (LI-RADS US-3) decreased with the assistance of the STARHE-DETECT model (20–23 *vs.* 24–31) while the number of detected lesions remained comparable (18–19/25), resulting in increased specificities (0.84–0.94) and positive likelihood ratios (4.75–12.00). There were no additional significant differences between interpretations with or without the assistance of the STARHE-DETECT model when stratified by the LI-RADS visualisation score, nodule size, and nodule echogenicity (Table S4). As expected, the detection performances decreased in VIS-C cine clips, HCCs ≤2 cm, and isoechoic HCCs for all the readers.

Discussion

In this prospective multicentric study, we developed a deep learning model for HCC risk stratification based on the analysis of the non-tumoral parenchyma on ultrasound (STARHE-RISK). This model could predict patients at high risk of developing an HCC with an odds ratio of 6.6, which is extremely promising for future risk-based personalised surveillance strategies. Moreover, the combination of the STARHE-RISK model and the FASTRAK score, a score combining routine clinical and biological parameters, outperformed both the STARHE-RISK model and the FASTRAK score, when considered separately, with an odds ratio of 8.9 to predict patients at high risk of HCC development. It also largely outperformed the radiologists' assessment, which was also subject to inter-reader variability (fair agreement). We also developed an object detection model that achieved robust performances in detecting early-stage HCC (mAP10 = 0.67), outperforming previously reported sensitivity of radiologists.[2,3] It is the first to be explicitly developed in patients with cACLD eligible for HCC surveillance, using ultrasound cine clips. With a 10% threshold for the intersection over the union between

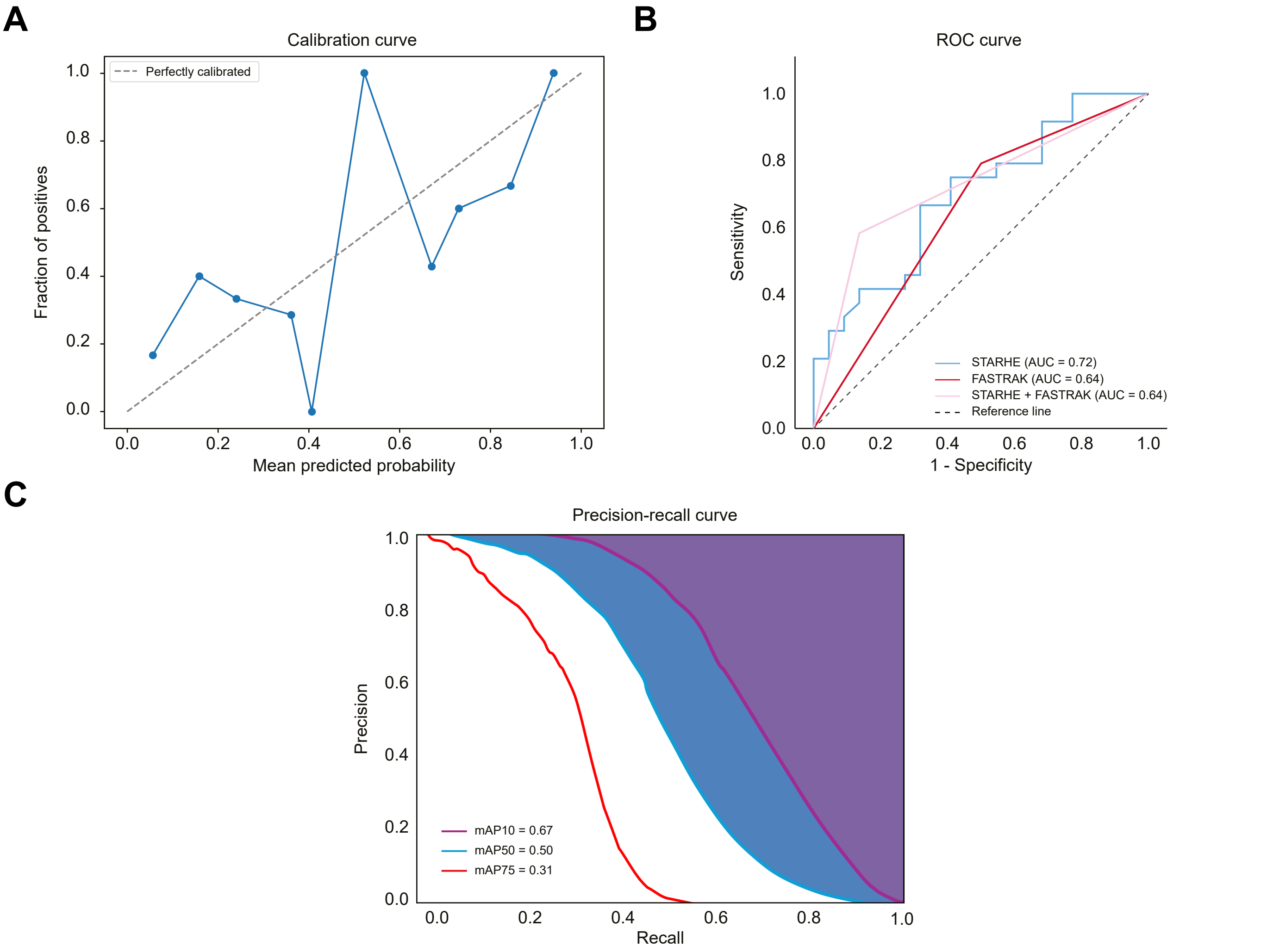

Fig. 2. Performance of STARHE-RISK and STARHE-DETECT models in the testing set. (**A**) Calibration curve and (**B**) ROC curve of the STARHE-RISK model and FASTRAK score for HCC risk stratification in the testing set. (**C**) Mean Average Precision (Intersection over Union of 10%, 50%, 75%) with precision-recall curve obtained by plotting the STARHE-DETECT model's precision and recall values as a function of the model's confidence score threshold.

the predicted box and the annotated box and a confidence level of 70%, the STARHE-DETECT model achieved an overall sensitivity of 0.68 and a sensitivity of 0.67 for HCCs ≤2 cm while maintaining few false positive predictions (14/75 [19%] cine clips with false positives and a median number of false positives of 2 [IQR 1–8] per video). The STARHE-DETECT model achieved detection performances comparable to three expert readers (sensitivity 0.68 *vs.* 0.76). Radiologist assistance with the STARHE-DETECT model increased the specificity and positive likelihood ratio of LI-RADS US-3 observations but not the sensitivity. The STARHE-DETECT model outperformed the reported sensitivity of ultrasound in the detection of early-stage (47%[2]) and very early-stage (22.5%[3]) HCC while maintaining comparable sensitivity (0.45) in ultrasound cine clips of poor quality (LI-RADS VIS-C). A key challenge in developing such deep learning models is achieving a balance between maximising the detection of early-stage HCCs and minimising the number of false positives. An excessive number of false positives could burden radiologists by requiring them to review and dismiss numerous findings, while also leading to unnecessary MRI referrals and increased pressure on healthcare systems. This balance is based on the optimal choice of the confidence level in the predicted boxes. A confidence level of 70% in the predicted box appeared to be the most clinically relevant as it achieved good performance with a sensitivity of 0.68 and few false positive predictions (median of 2 [IQR 1–8] in 14/75 cine clips [19%]). Moreover, incorporating prior ultrasound examinations into the model's predictions could help reduce the rate of false positives, as stable findings during surveillance could be excluded.

The solid multicentric methodology of the STARHE study reinforces these results. The HCC risk stratification model is the first prospective multicentric study aiming to develop such a model on ultrasound. The model relies on a simple, short (10 s), free breathing and standardised ultrasound acquisition, requiring no learning curve. In contrast, the STARHE-DETECT model was trained on ultrasound cine clips to develop a reliable clinical tool that can be easily integrated into clinical practice. Furthermore, we intended to include a

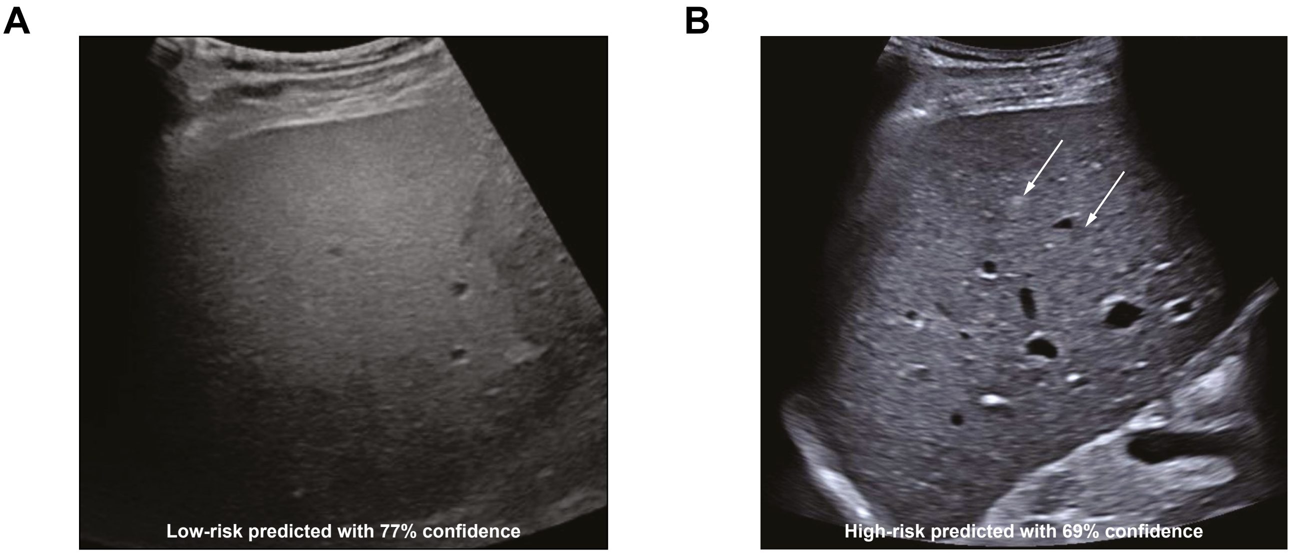

Fig. 3. Liver parenchyma echotexture patterns and HCC risk stratification performed by STARHE-RISK. (**A**) Increased homogeneous echostructure in a 55-year-old man with alcohol-related cirrhosis correctly predicted at low-risk (77% confidence) compared to (**B**) a macronodular echostructure (arrow) in a 42-year-old man with cured hepatitis B cirrhosis and BCLC 0 hepatocellular carcinoma (not shown on the image) correctly predicted at high-risk (on this view of the non-tumoral liver parenchyma).

Table 3. Performances of the deep learning object detection model for the detection of early-stage HCC (BCLC 0 or A).

	Rate of detected lesions	Rate of false positives with median - IQR per video)	Sensitivity	Specificity	Positive predictive value	Negative predictive value	Positive likelihood ratio	Negative likelihood ratio	Accuracy
**Deep learning model**	17/25 (68%)	14/75 (19%) 2 [1–8]	0.68 (0.47–0.85)	0.82 (0.69–0.91)	0.65 (0.50–0.78)	0.84 (0.74–0.90)	3.78 (1.97–7.24)	0.39 (0.22–0.70)	0.77 (0.66–0.86)
**LI-RADS visualisation score**									
VIS-A (n = 31–51)	NA	NA	0.73 (0.57–0.85)	0.85 (0.74–0.92)	0.74 (0.62–0.84)	0.84 (0.76–0.89)	4.76 (2.68–8.44)	0.32 (0.20–0.53)	0.80 (0.72–0.87)
VIS-B (n = 17–24)	NA	NA	0.70 (0.45–0.88)	0.79 (0.64–0.90)	0.61 (0.45–0.75)	0.85 (0.74–0.92)	3.34 (1.75–6.39)	0.38 (0.19–0.75)	0.76 (0.64–0.86)
VIS-C (n = 7–20)	NA	NA	0.45 (0.17–0.77)	0.80 (0.63–0.92)	0.42 (0.22–0.64)	0.82 (0.73–0.89)	2.27 (0.90–5.74)	0.68 (0.39–1.20)	0.72 (0.57–0.84)
**Nodule size**									
≤2.0 cm (n = 12)	8/12 (67%)	NA	0.67 (0.35–0.90)	0.82 (0.69–0.91)	0.47 (0.30–0.64)	0.91 (0.82–0.96)	3.70 (1.81–7.56)	0.41 (0.18–0.91)	0.79 (0.67–0.88)
2.0–3.0 cm (n = 8)	5/8 (63%)	NA	0.63 (0.24–0.91)	0.82 (0.69–0.91)	0.36 (0.20–0.55)	0.93 (0.85–0.97)	3.47 (1.56–7.72)	0.46 (0.19–1.13)	0.79 (0.67–0.89)
>3.0 cm (n = 5)	4/5 (80%)	NA	0.80 (0.28–0.99)	0.82 (0.69–0.91)	0.31 (0.18–0.48)	0.98 (0.69–1.00)	4.44 (2.13–9.28)	0.24 (0.04–1.41)	0.82 (0.69–0.91)
**Nodule echogenicity**									
Hypoechoic (n = 7)	6/7 (86%)	NA	0.86 (0.42–1.00)	0.82 (0.69–0.91)	0.40 (0.25–0.58)	0.98 (0.87–1.00)	4.76 (2.45–9.25)	0.17 (0.03–1.07)	0.83 (0.70–0.91)
Isoechoic (n = 8)	4/8 (50%)	NA	0.50 (0.16–0.84)	0.82 (0.69–0.91)	0.31 (0.15–0.53)	0.91 (0.84–0.95)	2.78 (1.12–6.91)	0.61 (0.30–1.23)	0.78 (0.65–0.87)
Hyperechoic (n = 6)	5/6 (83%)	NA	0.83 (0.36–1.00)	0.82 (0.69–0.91)	0.36 (0.22–0.53)	0.98 (0.87–1.00)	4.63 (2.32–9.24)	0.20 (0.03–1.22)	0.82 (0.70–0.91)
Heterogeneous (n = 4)	2/4 (50%)	NA	0.50 (0.07–0.93)	0.82 (0.69–0.91)	0.18 (0.07–0.41)	0.95 (0.88–0.98)	2.78 (0.69–8.73)	0.61 (0.23–1.64)	0.80 (0.66–0.89)

comprehensive representation of the very early and early stages of HCC on ultrasound, considering both size and echogenicity, to reinforce the robustness of the model's training. Indeed, some of the patients with ultrasound initially reported normal but positive AFP ultimately had a diagnosis of HCC on CT or MRI. These patients had a second-look ultrasound to meet the needs of the study.

The inclusion of patients eligible for HCC surveillance programs, the representation of the most common aetiologies of chronic liver disease (alcohol-related liver disease, metabolic dysfunction-associated steatotic liver disease, controlled HBV and cured HCV), and the use of ultrasound cine clips mimic the real-life practice of surveillance ultrasound, making the developed models applicable in clinical practice. Both models have

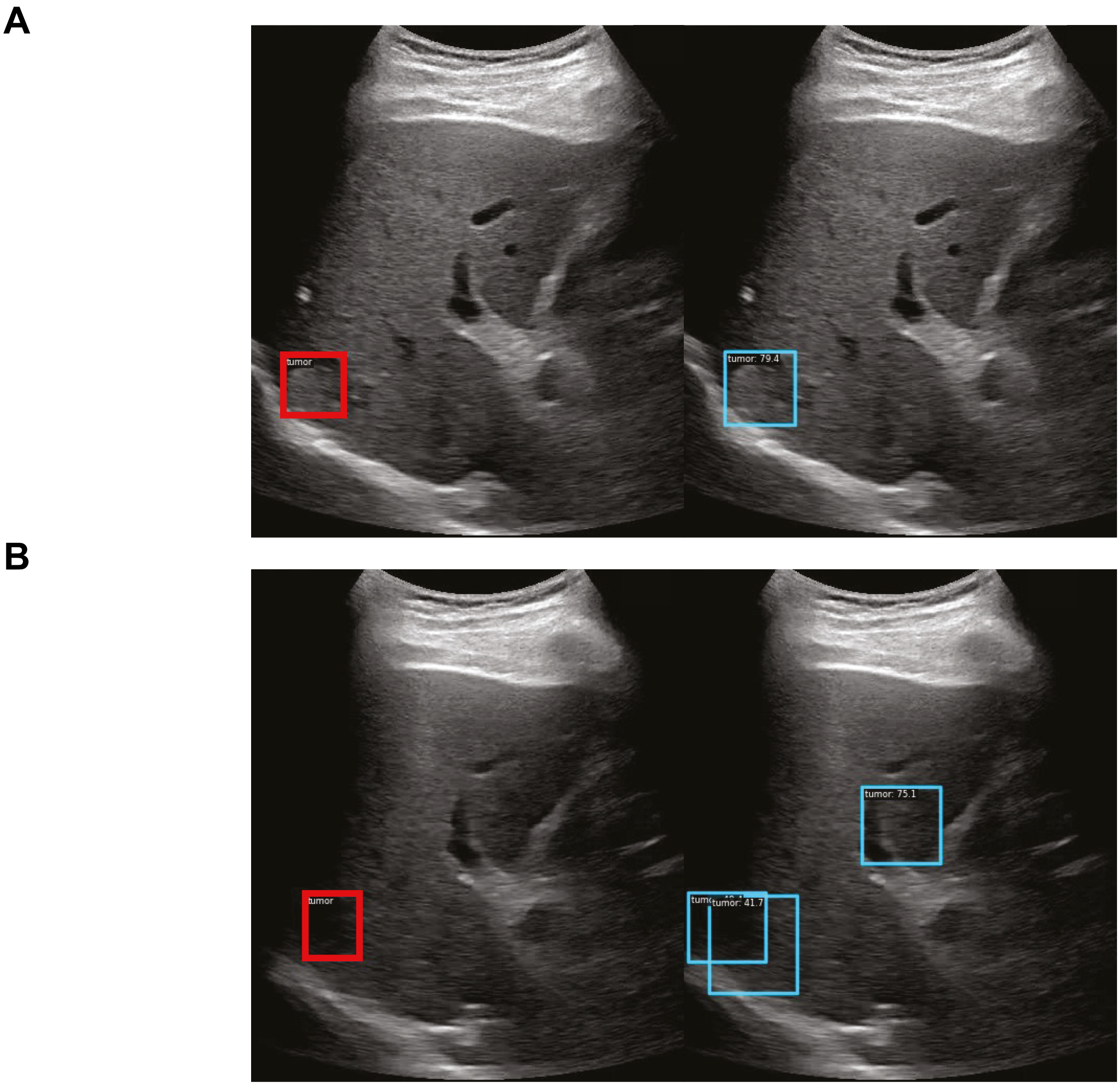

Fig. 4. Correctly detected HCC nodule and false positive prediction by STARHE-DETECT model in a 74-year-old patient with metabolic dysfunction-associated steatotic liver disease and 17 mm isoechoic hepatocellular carcinoma. The HCC nodule in **Figure 4A** was correctly detected with a confidence level of 79.4%. **Figure 4B** shows a false positive prediction in the non-tumoral parenchyma with a high confidence level (75%). In **Figure 4B**, the partially obscured lesion was detected with a lower confidence level (42%). Note: Red bounding boxes represent the manually annotated ground truth, and blue bounding boxes are the HCC nodules predicted by the deep learning model.

been developed following state-of-the-art artificial intelligence methodology with an independent testing set, stratified according to potential confounders including aetiology of liver disease, FASTRAK score, and ultrasound manufacturer. In addition, the testing set was designed to be representative of the targeted population with upstream sample size calculation based on previous reports.[24–26] However, the testing set did not reflect the real-life prevalence of HCC, which might have resulted in overestimated positive and negative predictive values. Finally, including two ultrasound manufacturers reinforces the generalisability of the models.

Regarding the STARHE-RISK model, the main limitation is the potential inclusion of patients at high risk of developing HCC in the control group. Indeed, although we intended to mitigate this potential bias with a follow-up at 1 year, a few patients at high risk of HCC development might have been included in the control group and may have developed an HCC after the 1-year follow-up. An alternative approach could have been to include patients without HCC at baseline and follow them longitudinally for several years. However, implementing this approach would have been highly challenging from clinical and deep learning perspectives (*e.g.* class imbalance between cases and controls). Also, to simplify the study design, there was no matched pair case-control, but this did not result in demographic group imbalance. The STARHE-RISK model was solely trained on the non-tumoral liver parenchyma, preventing any biased training from the tumour itself. In addition, only patients with early-stage HCC were included, none with more advanced stages, to prevent the inclusion of patients with infiltrative HCC. However, testing the model on the non-tumoral parenchyma of patients with early-stage HCC could have theoretically overestimated the model's performance compared with patients without HCC at the time of inclusion.

Table 4. Performances of the three radiologists (reader 1 with 8 years of experience, reader 2 with 6 years of experience and reader 3 with 35 years of experience) without and with the assistance of the deep learning object detection model for the detection of early-stage HCC (BCLC 0 or A).

	LI-RADS US-3 observations	Rate of detected lesions	Sensitivity	Specificity	Positive predictive value	Negative predictive value	Positive likelihood ratio	Negative likelihood ratio	Accuracy
**Radiologists alone**									
Pooled performances	NA	NA	0.76 (0.65–0.85)	0.86 (0.79–0.91)	0.73 (0.64–0.80)	0.88 (0.83–0.92)	5.43 (3.58–8.23)	0.28 (0.19–0.42)	0.83 (0.77–0.87)
Reader 1	31	19/25 (76%)	0.76 (0.55–0.91)	0.76 (0.62–0.87)	0.61 (0.48–0.73)	0.86 (0.76–0.83)	3.17 (1.84–5.44)	0.32 (0.15–0.65)	0.76 (0.65–0.85)
Reader 2	23	19/25 (76%)	0.76 (0.55–0.91)	0.92 (0.81–0.98)	0.83 (0.64–0.93)	0.88 (0.79–0.94)	9.50 (3.62–24.95)	0.26 (0.13–0.53)	0.87 (0.77–0.93)
Reader 3	24	19/25 (76%)	0.76 (0.55–0.91)	0.88 (0.76–0.95)	0.76 (0.59–0.87)	0.88 (0.78–0.94)	6.33 (2.90–13.85)	0.27 (0.13–0.55)	0.84 (0.74–0.91)
**Radiologists with the assistance of the deep learning model**									
Pooled performances	NA	NA	0.75 (0.63–0.84)	0.90 (0.94–0.94)	0.79 (0.69–0.86)	0.88 (0.83–0.91)	7.47 (4.54–12.28)	0.28 (0.19–0.42)	0.85 (0.80–0.89)
Reader 1	21	18/25 (72%)	0.72 (0.51–0.88)	0.94 (0.84–0.99)	0.86 (0.66–0.95)	0.87 (0.78–0.93)	12.00 (3.90–36.93)	0.30 (0.16–0.56)	0.87 (0.77–0.93)
Reader 2	27	19/25 (76%)	0.76 (0.55–0.91)	0.84 (0.71–0.93)	0.70 (0.55–0.82)	0.88 (0.78–0.93)	4.75 (2.43–9.30)	0.29 (0.14–0.58)	0.81 (0.71–0.89)
Reader 3	23	19/25 (76%)	0.76 (0.55–0.91)	0.92 (0.81–0.98)	0.83 (0.64–0.93)	0.88 (0.79–0.94)	9.50 (3.62–24.95)	0.26 (0.13–0.53)	0.87 (0.77–0.93)

Finally, the size of the testing dataset did not allow us to perform reliable subgroup analysis based on the aetiology of the cACLD. However, universal scoring systems are more suitable in clinical practice, as patients usually cumulate several causes of chronic liver diseases.

Regarding the STARHE-DETECT model, the main limitation of the study to compare its performance alongside radiologists' reading is the difference in the prevalence of HCC in the test set (33% of the cine clips) compared with clinical practice (annual incidence <3%). Although the readers were unaware of the prevalence of HCC in the test set, it was easy to suspect that this study required a higher prevalence than that of routine clinical practice. However, the median HCC size (2.5 cm) was consistent with reported data in surveillance programs.[42] Another limitation is the lack of prospective implementation of the STARHE-DETECT model alongside the performance of the ultrasound examination by radiologists or sonographers. In fact, although minimised by the strict methodology, the retrospective reading of ultrasound cine clips may have led to an overestimation of the detection performance of the expert readers, while it can be hypothesised that it less affected the performance of a deep learning model. In clinical practice, a significant limitation of ultrasound is the risk of the sonographer overlooking certain liver regions too quickly, which should have less impact on a deep learning model analysing each image separately. The lack of prospective implementation of the detection model precludes any conclusion on the rate of false positive LI-RADS US-3 observations in real-life ultrasound examinations, which are typically much longer than the short cine clips analysed in this study. Additionally, future randomised studies evaluating the STARHE-DETECT model should account for the experience level of the sonographer/radiologist and consider the varying implementation of HCC ultrasound surveillance in Europe (primarily performed by radiologists) and North America (performed by autonomous sonographers).

This study paves the way for a personalised surveillance program based on the predicted risk of HCC development using liver imaging. Alongside simple algorithms combining clinical and biological routine parameters such as the FAS-TRAK score, the STARHE-RISK model will provide an urgently needed tool for personalised HCC surveillance, which will not ultimately rely on a single test but rather on a combination of approaches mixing clinical, biological, and radiological data. The use of ultrasound to develop this model also reinforces its applicability given its cornerstone position in the diagnosis of cACLD, and its availability. Moreover, by improving ultrasound surveillance performance and identifying patients at very high risk of HCC development, both models will significantly contribute to increasing the number of patients with an HCC detected at an early stage, making them eligible for curative treatment with a better prognosis. Improving ultrasound surveillance performance for early-stage HCC would also redefine the cost-effectiveness studies on HCC surveillance.[13–16,19,31]

The next step will be to design and prospectively validate a risk stratification-based personalised surveillance strategy integrating clinical, blood, and imaging risk stratification scores and relying on abbreviated MRI for patients at high risk of HCC development. This paradigm shift could have a considerable positive impact on the quality of patient care and is currently

tested in risk-based randomised trials.[31] Such a strategy could be refined over time with the discovery of new blood biomarkers. In addition, both models will likely improve over the coming years by including new patients in longitudinal follow-up cohorts. These longitudinal cohorts will allow us to calculate individual annual HCC risk for each patient.

In conclusion, the STARHE-RISK model is a robust ultrasound-based deep learning model that stratifies the risk of HCC development based on a simple, short (10 s), free breathing, and standardised ultrasound acquisition. The combination of the STARHE-RISK model and the FASTRAK score outperformed both the STARHE-RISK model and the FASTRAK score when considered separately. The STARHE-DETECT model demonstrated strong potential for detecting early-stage HCC and could become a valuable surveillance tool to assist radiologists and sonographers in surveillance.

This study paves the way for a risk-based personalised surveillance program that will not ultimately rely on a single test but rather on a combination of approaches mixing clinical, biological, and radiological data. Future prospective studies are needed to validate the STARHE-RISK model and risk-based personalised surveillance programmes in longitudinal cohorts of patients with cACLD without HCC at inclusion with real-life HCC prevalence, and to assess the STARHE-DETECT model's performance alongside radiologists' reading in an integrated workflow.[43]

Affiliations

[1]*Université de Strasbourg, Inserm, UMRS 1110, Institute for Translational Medicine and Liver Disease, Strasbourg, France;* [2]*Institute of Image-Guided Surgery, IHU Strasbourg, Strasbourg, France;* [3]*Department of Diagnostic Radiology, McGill University, Montreal, Canada;* [4]*Augmented Intelligence & Precision Health Laboratory (AIPHL), McGill University Health Centre Research Institute, Montreal, Canada;* [5]*ICube, Laboratory of Engineering, Computer Science and Imaging, Department of Robotics, Imaging, Teledetection and Healthcare Technologies, University of Strasbourg, CNRS, UMR 7357, Strasbourg, France;* [6]*Department of Radiology, CHU Angers, Université d'Angers, Angers, France;* [7]*Laboratoire HIFIH UPRES EA3859, SFR ICAT 4208, Angers University, Université d'Angers, Angers, France;* [8]*Department of Radiology, Hôpital de la Croix Rousse, Hospices Civils de Lyon, Lyon, France;* [9]*Department of Radiology, APHP.Nord, Clichy, France;* [10]*Université Paris Cité, CRI UMR1149, Paris, France;* [11]*Interventional Radiology Unit, Hôpitaux Universitaires Paris Seine Saint-Denis, AP-HP, Bobigny, France;* [12]*Institut de Cancérologie de Montpellier, Montpellier, France;* [13]*Department of Diagnostic and Interventional Radiology, Hôpital Edouard Herriot, Hospices Civils de Lyon, Lyon, France;* [14]*Université de Lyon, LabTAU - INSERM U1032, Lyon, France;* [15]*FHU MOSAIC, Université Paris Cité, Paris, France;* [16]*Department of Diagnostic and Interventional Radiology, Hôpital Saint Eloi, CHU Montpellier, Montpellier, France;* [17]*Service d'Hépatologie, Hôpital de la Croix Rousse Hospices Civils de Lyon, Lyon, France;* [18]*Pôle Hépato-digestif, Service d'Hépatogastroentérologie, Hôpitaux Universitaires de Strasbourg, Strasbourg, France;* [19]*Department of Radiology, American Hospital of Paris, Paris, France;* [20]*Inria, Institut national de recherche en sciences et technologies du numérique, Paris, France;* [21]*Liver Unit, Hôpitaux Universitaires Paris Seine Saint-Denis, AP-HP, Bobigny, France;* [22]*Université Sorbonne Paris Nord, F-93000 Bobigny, France;* [23]*Inserm, UMR-1138 "Functional Genomics of Solid Tumors", Centre de Recherche des Cordeliers, Université de Paris, Paris, France*

Abbreviations

AFP, α-foetoprotein; AI, autoimmune; ALD, alcohol-related liver disease; ALT, alanine aminotransferase; AST, aspartate aminotransferase; BCLC, Barcelona-Clinic Liver Cancer; cACLD, compensated advanced chronic liver disease; CT, computed tomography; GGT, gamma-glutamyl transferase; HCC, hepatocellular carcinoma; INR, international normalised ratio; IQR, interquartile range; LI-RADS, Liver Imaging Reporting And Data System; mAP, mean average precision; MASLD; metabolic dysfunction-associated steatotic liver disease; MRI, magnetic resonance imaging.

Financial support

This work was supported by the French National Research Agency within the France 2030 program (ANR-21-RHUS-0001 DELIVER – TFB, PN) and within the Plan Investissements d'Avenir (ANR-10-IAHU-02 – TFB). PN's research is funded in part by the European Union (GENIAL, Grant agreement ID: 101096312) and by France 2030 RHU LIVER-TRACK (ANR-23-RHUS-0014). TB acknowledges funding by the European Union ERC-AdG-2020-FIBCAN #101021417, the Foundation of the University of Strasbourg, and the French state funds managed within the ANR (ANR-10-LABX-0028). This work of the Interdisciplinary Thematic Institute IMCBio, as part of the ITI 2021-2028 program of the University of Strasbourg, CNRS and Inserm, was supported by IdEx Unistra (ANR-10-IDEX-0002), and by SFRI-STRAT'US project (ANR 20-SFRI-0012) and EUR IMCBio (ANR-17-EURE-0023) under the framework of the French Investments for the Future Program.

Conflicts of interest

MR received speaker fees from Terumo, Guerbet, Sirtex, General Electrics, Servier, and Canon. PN has received honoraria from and/or consults for AstraZeneca, Bayer, Bristol-Myers Squibb, Eisai, Gilead, Guerbet, Ipsen, and Roche. He received research grants from AstraZeneca, AbbVie, Bristol-Myers Squibb and Eisai. TFB is founder, shareholder, and advisor and received research grant support from Alentis Therapeutics. He serves also as advisor and consultant to Pueros Bioventures and Novo Holding. The other authors have no conflicts of interest to declare.

Please refer to the accompanying ICMJE disclosure forms for further details.

Authors' contributions

Substantial contributions to the conception, design, and implementation of the study: JD, BG, TFB, NP, CS, VV, MR, PN, AT. Acquisition of the data: AP, AR, RS, OS, CC, LM, JG, JL, VS. Analysis of the data: JD, AM, J-PM, NP. Interpretation of the data: JD, AM, NP, JL, VV, MR, PN, BG, TFB.

Data availability

Data may be requested from the corresponding author (JD – IHU Strasbourg, Strasbourg, France).

Acknowledgements

Elsa Malet, Kahina Saichi, Laura Roth, and Pierre Goepfert from the IHU Strasbourg clinical trial team. Marie Bost from the CHU Angers clinical trial team. Sébastien Garnier from the CHU Lyon – Croix Rousse Hospital clinical trial team. Slimane Bendou from the APHP – Beaujon Hospital clinical trial team. Tahira Hussain from the APHP – Avicenne Hospital clinical trial team. Chloé Guillot from the CHU Montpellier – Saint Eloi Hospital clinical trial team. Biljana Hadzic from the CHU Lyon – Edouard Herriot Hospital clinical trial team.

Supplementary data

Supplementary data to this article can be found online at https://doi.org/10.1016/j.jhepr.2025.101510.

References

Author names in bold designate shared co-first authorship

[1] Rumgay H, Arnold M, Ferlay J, et al. Global burden of primary liver cancer in 2020 and predictions to 2040. J Hepatol 2022;77:1598–1606.

[2] **Tzartzeva K**, **Obi J**, Rich NE, et al. Surveillance imaging and alpha fetoprotein for early detection of hepatocellular carcinoma in patients with cirrhosis: a meta-analysis. Gastroenterology 2018;154:1706–1718.e1701.

[3] **Park HJ**, **Jang HY**, Kim SY, et al. Non-enhanced magnetic resonance imaging as a surveillance tool for hepatocellular carcinoma: comparison with ultrasound. J Hepatol 2020;72:718–724.

[4] Goutté N, Sogni P, Bendersky N, et al. Geographical variations in incidence, management and survival of hepatocellular carcinoma in a Western country. J Hepatol 2017;66:537–544.
[5] Nahon P, Najean M, Layese R, et al. Early hepatocellular carcinoma detection using magnetic resonance imaging is cost-effective in high-risk patients with cirrhosis. JHEP Rep 2022;4:100390.
[6] Gupta P, Soundararajan R, Patel A, et al. Abbreviated MRI for hepatocellular carcinoma screening: a systematic review and meta-analysis. J Hepatol 2021;75:108–119.
[7] Chan MV, Huo YR, Trieu N, et al. Noncontrast MRI for hepatocellular carcinoma detection: a systematic review and meta-analysis - a potential surveillance tool? Clin Gastroenterol Hepatol 2022;20:44–56.e42.
[8] Kim DH, Choi SH, Shim JH, et al. Meta-analysis of the accuracy of abbreviated magnetic resonance imaging for hepatocellular carcinoma surveillance: non-contrast versus hepatobiliary phase-abbreviated magnetic resonance imaging. Cancers 2021;13:2975.
[9] Vietti Violi N, Lewis S, Liao J, et al. Gadoxetate-enhanced abbreviated MRI is highly accurate for hepatocellular carcinoma screening. Eur Radiol 2020;30:6003–6013.
[10] Khatri G, Pedrosa I, Ananthakrishnan L, et al. Abbreviated-protocol screening MRI vs. complete-protocol diagnostic MRI for detection of hepatocellular carcinoma in patients with cirrhosis: an equivalence study using LI-RADS v2018. J Magn Reson Imaging 2020;51:415–425.
[11] Besa C, Lewis S, Pandharipande PV, et al. Hepatocellular carcinoma detection: diagnostic performance of a simulated abbreviated MRI protocol combining diffusion-weighted and T1-weighted imaging at the delayed phase post gadoxetic acid. Abdom Radiol (NY) 2017;42:179–190.
[12] Park MS, Kim S, Patel J, et al. Hepatocellular carcinoma: detection with diffusion-weighted versus contrast-enhanced magnetic resonance imaging in pretransplant patients. Hepatology 2012;56:140–148.
[13] Kim HL, An J, Park JA, et al. Magnetic resonance imaging is cost-effective for hepatocellular carcinoma surveillance in high-risk patients with cirrhosis. Hepatology 2019;69:1599–1613.
[14] Ronot M, Nahon P, Rimola J. Screening of liver cancer with abbreviated MRI. Hepatology 2023;78:670–686.
[15] Semmler G, Meyer EL, Kozbial K, et al. HCC risk stratification after cure of hepatitis C in patients with compensated advanced chronic liver disease. J Hepatol 2022;76:812–821.
[16] Ioannou GN, Green P, Kerr KF, et al. Models estimating risk of hepatocellular carcinoma in patients with alcohol or NAFLD-related cirrhosis for risk stratification. J Hepatol 2019;71:523–533.
[17] Innes H, Jepsen P, McDonald S, et al. Performance of models to predict hepatocellular carcinoma risk among UK patients with cirrhosis and cured HCV infection. JHEP Rep 2021;3:100384.
[18] Audureau E, Carrat F, Layese R, et al. Personalized surveillance for hepatocellular carcinoma in cirrhosis - using machine learning adapted to HCV status. J Hepatol 2020;73:1434–1445.
[19] **Singal AG**, **Sanduzzi-Zamparelli M**, Nahon P, et al. International Liver Cancer Association (ILCA) white paper on hepatocellular carcinoma risk stratification and surveillance. J Hepatol 2023;79:226–239.
[20] Fujiwara N, Kobayashi M, Fobar AJ, et al. A blood-based prognostic liver secretome signature and long-term hepatocellular carcinoma risk in advanced liver fibrosis. Med 2021;2:836–850.e810.
[21] Hoshida Y, Villanueva A, Sangiovanni A, et al. Prognostic gene expression signature for patients with hepatitis C–related early-stage cirrhosis. Gastroenterology 2013;144:1024–1030.
[22] Fujiwara N, Kubota N, Crouchet E, et al. Molecular signatures of long-term hepatocellular carcinoma risk in nonalcoholic fatty liver disease. Sci Transl Med 2022;14:eabo4474.
[23] Nahon P, Bamba-Funck J, Layese R, et al. Integrating genetic variants into clinical models for hepatocellular carcinoma risk stratification in cirrhosis. J Hepatol 2023;78:584–595.
[24] Kitamura S, Iishi H, Tatsuta M, et al. Liver with hypoechoic nodular pattern as a risk factor for hepatocellular carcinoma. Gastroenterology 1995;108:1778–1784.
[25] Tarao K, Hoshino H, Shimizu A, et al. Patients with ultrasonic coarse-nodular cirrhosis who are anti-hepatitis C virus-positive are at high risk for hepatocellular carcinoma. Cancer 1995;75:1255–1262.
[26] Caturelli E, Castellano L, Fusilli S, et al. Coarse nodular US pattern in hepatic cirrhosis: risk for hepatocellular carcinoma. Radiology 2003;226:691–697.
[27] Hickman SE, Payne NR, Black RT, et al. Mammography breast cancer screening triage using deep learning: a UK retrospective study. Radiology 2023;309:e231173.
[28] Cheng PM, Montagnon E, Yamashita R, et al. Deep learning: an update for radiologists. RadioGraphics 2021;41:1427–1445.
[29] Reig M, Forner A, Rimola J, et al. BCLC strategy for prognosis prediction and treatment recommendation: the 2022 update. J Hepatol 2022;76:681–693.
[30] Ferraioli G, Barr RG, Berzigotti A, et al. WFUMB guideline/guidance on liver multiparametric ultrasound: part 1. Update to 2018 guidelines on liver ultrasound elastography. Ultrasound Med Biol 2024;50:1071–1087.
[31] Nahon P, Ronot M, Sutter O, et al. Study protocol for FASTRAK: a randomised controlled trial evaluating the cost impact and effectiveness of FAST-MRI for HCC suRveillance in pAtients with high risK of liver cancer. BMJ Open 2024;14:e083701.
[32] Mazellier J-P, Boujon A, Bour-Lang M, et al. MOSaiC: a web-based platform for collaborative medical video assessment and annotation. arXiv 2023. https://doi.org/10.48550/arXiv.2312.08593. preprint.
[33] Bradshaw TJ, Huemann Z, Hu J, et al. A guide to cross-validation for artificial intelligence in medical imaging. Radiol Artif Intell 2023;5:e220232.
[34] Fan H, Xiong B, Mangalam K, et al. Multiscale vision transformers. In: Proceedings of the IEEE/CVF international conference on computer vision; 2021. p. 6824–6835.
[35] Tran D, Bourdev L, Fergus R, et al. Learning spatiotemporal features with 3d convolutional networks. In: Proceedings of the IEEE international conference on computer vision; 2015. p. 4489–4497.
[36] Carreira J, Zisserman A. Quo vadis, action recognition? A new model and the kinetics dataset. In: Proceedings of the IEEE conference on computer vision and pattern recognition; 2017. p. 6299–6308.
[37] Kay W, Carreira J, Simonyan K, et al. The kinetics human action video dataset. arXiv 2017. https://doi.org/10.48550/arXiv.1705.06950. preprint.
[38] Chattopadhay A, Sarkar A, Howlader P, et al. Grad-cam++: generalized gradient-based visual explanations for deep convolutional networks. In: 2018 IEEE winter conference on applications of computer vision (WACV). IEEE; 2018. p. 839–847.
[39] LI-RADS® ultrasound surveillance v2024 Core. American College of Radiology; 2024.
[40] Fleiss JL, Levin B, Paik MC. Statistical methods for rates and proportions. Wiley: Wiley Series in Probability and Statistics; 2004.
[41] Landis JR, Koch GG. The measurement of observer agreement for categorical data. Biometrics 1977;33:159–174.
[42] Miquel M, Sopeña J, Vergara M, et al. Factors related to survival in hepatocellular carcinoma in the geographic area of Sabadell (Catalonia, Spain). Rev Esp Enferm Dig 2012;104:242.
[43] Ying H, Liu X, Zhang M, et al. A multicenter clinical AI system study for detection and diagnosis of focal liver lesions. Nat Commun 2024;15:1131.

Keywords: Hepatocellular carcinoma; Risk stratification; Prediction; Ultrasound; Deep learning.
Received 11 March 2025; received in revised form 14 June 2025; accepted 30 June 2025; Available online 5 July 2025

Journal of Hepatology, Volume *7*

Supplemental information

Improving risk stratification and detection of early HCC using ultrasound-based deep learning models

Jérémy Dana, Adrien Meyer, Anita Paisant, Agnès Rode, Riccardo Sartoris, Olivier Séror, Christophe Cassinotto, Laurent Milot, Jules Grégory, Jules Cœur, Jérôme Lebigot, Valentina Schembri, François Villeret, Armelle-Natsuo Takeda, Maxime Ronot, Valérie Vilgrain, Thomas F. Baumert, Benoit Gallix, Nicolas Padoy, and Pierre Nahon

Improving risk stratification and detection of early HCC using ultrasound-based deep learning models

Jérémy Dana, Adrien Meyer, Anita Paisant, Agnès Rode, Riccardo Sartoris, Olivier Séror, Christophe Cassinotto, Laurent Milot, Jules Grégory, Jules Cœur, Jérôme Lebigot, Valentina Schembri, François Villeret, Armelle-Natsuo Takeda, Maxime Ronot, Valérie Vilgrain, Thomas F Baumert, Benoit Gallix, Nicolas Padoy, Pierre Nahon

Table of contents

Supplementary methods…………………………………………………………….... 2

Fig. S1……………………………………………………………………………….. 3

Fig. S2……………………………………………………………………………….. 3

Table S1……………………………………………………………………………...4

Table S2……………………………………………………………………………...4

Table S3……………………………………………………………………………...4

Table S4……………………………………………………………………………...5

Supplementary methods

Performance in Simulated Low-Prevalence Setting

Considering the diagnostic performance of the STARHE-RISK model in the testing set and the estimated real-life prevalence of HCC (3%), the simulated positive and negative predictive values are 7.4% and 98.8% for STARHE-RISK, 4.7% and 98.7% for FASTRAK, 11.4% and 98.5% for STARHE-RISK combined with FASTRAK, respectively. Regarding STARHE-DETECT, the simulated positive and negative predictive values were 10.5% and 98.8%, respectively.

Description of models' architecture for STARHE-RISK and STARHE-DETECT models

The C3D model is a 3D convolutional neural network designed for video input. It processes video clips using eight 3×3×3 convolutional layers with ReLU activation, interspersed with five 3D max-pooling layers. The output is then passed through two fully connected layers of 4,096 units each, followed by a softmax classifier for final prediction.

RTMDet is a one-stage, anchor-free object detector optimized for real-time performance. It strikes a balance between speed and accuracy using a streamlined CSP-Darknet backbone and a neck composed of large-kernel depth-wise convolutions to enhance feature representation. A two-layer regression head predicts bounding boxes, while a linear classification head assigns class labels. During training, dynamic soft-label assignment is used to address class imbalance effectively.

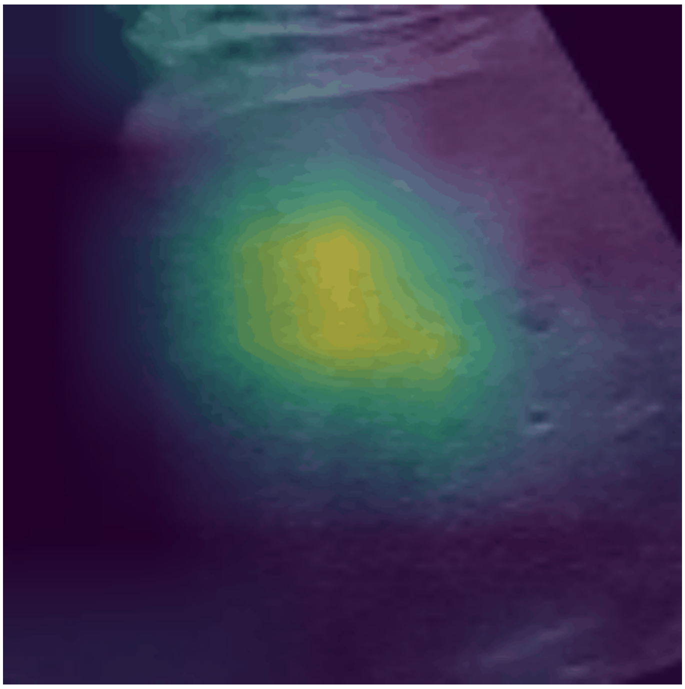

Fig. S1 – Example of Grad-CAM++ explainability map for the STARHE-RISK model demonstrating that the region of interest lies in the liver parenchyma.

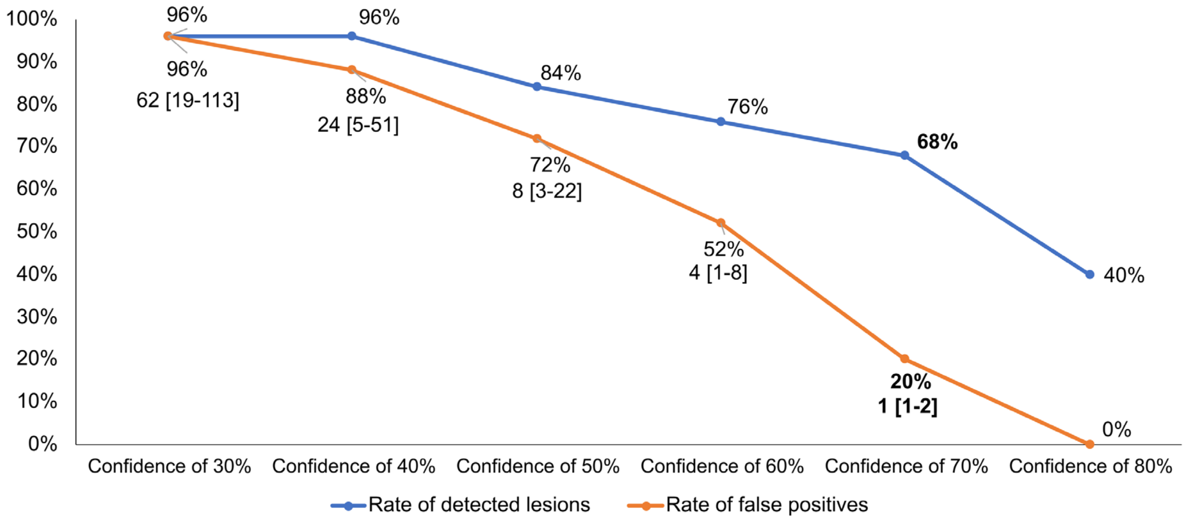

Fig. S2 – Rates of detected HCC nodules and false positives (with the median number of false positives and interquartile range per video) by the deep learning detection model on the 25 B-mode HCC ultrasound cine clips across different confidence levels in the predicted bounding box. A confidence level of 70% appeared to be the most clinically relevant as it achieved good performances with a rate of detected lesions of 68% (17/25).

	Model and hyperparameters	Final model
**Batch sizes**	2, 4, 8, 16, 32	4
**Learning rate**	0.000005, 0.00001, 0.000015, 0.00002, 0.000025, 0.00003, 0.0000375, 0.00005, 0.0001, 0.0016	45 epochs with learning rate of 0.00002 (divided by 10 at 20 and 40 epochs)
**Optimizer**	SGD, AdamW	SGD
**Input/output size**	128x128, 256x256	128x128
**Transfer learning**	All except prediction head	All except prediction head
**Models**	MViT, C3D, I3D	C3D

Table S1 - Model and hyperparameters selection for STARHE-RISK model.

	Model and hyperparameters	Final model
**Batch sizes**	2, 4, 8, 16	8
**Learning rate**	0.0008, 0.0006, 0.0004, 0.0002, 0.0001, 0.001, 0.002, 0.005	45 epochs with learning rate of 0.0002 (divided by 10 at 20 and 40 epochs)
**Frozen stages**	-1, 0, 1	-1 (none)
**Batch requires grads**	True, False	True
**Transfer learning**	All except prediction head	All except prediction head
**Models**	Faster-RCNN, DINO-DETR, RTMDet	RTMDet

Table S2 - Model and hyperparameters selection for STARHE-DETECT model

	Sensitivity	Specificity	Positive predictive value	Negative predictive value	Accuracy
VIS-A	0.80 [0.65-0.90]	0.48 [0.29-0.68]	0.72 [0.63-0.79]	0.59 [0.42-0.74]	0.68 [0.56-0.79]
VIS-B	0.67 [0.43-0.85]	0.73 [0.50-0.89]	0.70 [0.53-0.83]	0.70 [0.54-0.82]	0.70 [0.54-0.83]
VIS-C	0.44 [0.14-0.79]	0.88 [0.70-0.98]	0.57 [0.27-0.83]	0.82 [0.72-0.89]	0.77 [0.60-0.90]

Table S3 – Pooled prediction performances of the STARHE-RISK classification model according to the LI-RADS visualisation score. Notes: 95% confidence in squared brackets.

	LI-RADS US-3 observations	Rate of detected lesions	Sensitivity	Specificity	Positive predicted value	Negative predictive value	Positive likelihood ratio	Negative likelihood ratio	Accuracy
**Reader 1 without deep learning assistance**	31	19/25 (76%)	0.76 (0.55-0.91)	0.76 (0.62-0.87)	0.61 (0.48-0.73)	0.86 (0.76-0.83)	3.17 (1.84-5.44)	0.32 (0.15-0.65)	0.76 (0.65-0.85)
**LI-RADS visualisation score**									
VIS-A (n = 51)	20	15/19 (79%)	0.79 (0.54-0.94)	0.84 (0.67-0.95)	0.75 (0.56-0.87)	0.87 (0.74-0.94)	5.05 (2.19-11.68)	0.25 (0.10-0.60)	0.82 (0.69-0.92)
VIS-B (n = 17)	8	3/4 (75%)	0.75 (0.19-0.99)	0.62 (0.32-0.86)	0.38 (0.20-0.59)	0.89 (0.58-0.98)	1.95 (0.80-4.75)	0.41 (0.07-2.34)	0.65 (0.38-0.86)
VIS-C (n = 7)	3	1/2 (50%)	0.50 (0.01-0.99)	0.60 (0.15-0.95)	0.33 (0.08-0.74)	0.75 (0.39-0.93)	1.25 (0.22-7.22)	0.83 (0.18-3.96)	0.57 (0.18-0.90)
**Nodule size**									
≤ 2.0 cm (n = 12)	20	8/12 (67%)	0.67 (0.35-0.90)	0.76 (0.62-0.87)	0.40 (0.26-0.56)	0.90 (0.81-0.96)	2.78 (1.47-5.24)	0.44 (0.19-0.99)	0.74 (0.62-0.84)
2.0-3.0 cm (n = 8)	18	6/8 (75%)	0.75 (0.35-0.97)	0.76 (0.62-0.87)	0.33 (0.21-0.49)	0.95 (0.85-0.98)	3.12 (1.66-5.90)	0.33 (0.10-1.10)	0.76 (0.63-0.86)
> 3.0 cm (n = 5)	17	5/5 (100%)	1.00 (0.48-1.00)	0.76 (0.61-0.87)	0.29 (0.20-0.41)	1.00 (0.91-1.00)	4.17 (2.54-6.82)	0.00 (NA)	0.78 (0.65-0.88)
**Nodule echogenicity**									
Hypoechoic (n = 7)	18	6/7 (86%)	0.86 (0.42-1.00)	0.76 (0.62-0.87)	0.33 (0.22-0.47)	0.97 (0.86-1.00)	3.57 (2.00-6.37)	0.19 (0.03-1.16)	0.77 (0.64-0.87)
Isoechoic (n = 8)	15	3/8 (38%)	0.38 (0.09-0.76)	0.76 (0.62-0.87)	0.20 (0.08-0.41)	0.88 (0.81-0.93)	1.56 (0.56-4.34)	0.82 (0.47-1.44)	0.71 (0.57-0.82)
Hyperechoic (n = 6)	18	6/6 (100%)	1.00 (0.54-1.00)	0.76 (0.62-0.87)	0.33 (0.23-0.45)	1.00 (0.91-1.00)	4.17 (2.54-6.82)	0.00 (NA)	0.79 (0.66-0.88)
Heterogeneous (n = 4)	16	4/4 (100%)	1.00 (0.40-1.00)	0.76 (0.62-0.87)	0.25 (0.17-0.35)	1.00 (0.91-1.00)	4.17 (2.54-6.82)	0.00 (NA)	0.78 (0.64-0.88)
**Reader 1 with deep learning assistance**	21	18/25 (72%)	0.72 (0.51-0.88)	0.94 (0.83-0.99)	0.86 (0.66-0.95)	0.87 (0.78-0.93)	12.00 (3.90-36.93)	0.30 (0.16-0.56)	0.87 (0.77-0.93)

**LI-RADS visualisation score**									
VIS-A (n = 51)	17	15/19 (79%)	0.79 (0.54-0.94)	0.94 (0.79-0.99)	0.88 (0.66-0.97)	0.88 (0.76-0.95)	12.6 (3.24-49.31)	0.22 (0.09-0.54)	0.88 (0.76-0.96)
VIS-B (n = 17)	3	2/4 (50%)	0.50 (0.07-0.93)	0.92 (0.64-1.00)	0.67 (0.19-0.94)	0.86 (0.69-0.94)	6.5 (0.78-54.31)	0.54 (0.20-1.46)	0.82 (0.57-0.96)
VIS-C (n = 7)	1	1/2 (50%)	0.50 (0.01-0.99)	1.00 (0.48-1.00)	1.00 (0.03-1.00)	0.83 (0.56-0.95)	NA	0.5 (0.13-2.00)	0.86 (0.42-1.00)
**Nodule size**									
≤ 2.0 cm (n = 12)	10	7/12 (58%)	0.58 (0.28-0.85)	0.94 (0.83-0.99)	0.70 (0.41-0.89)	0.90 (0.83-0.85)	9.72 (2.94-32.18)	0.44 (0.23-0.87)	0.87 (0.76-0.94)
2.0-3.0 cm (n = 8)	9	6/8 (75%)	0.75 (0.35-0.97)	0.94 (0.83-0.99)	0.67 (0.38-0.87)	0.86 (0.88-0.99)	12.5 (3.89-40.19)	0.27 (0.08-0.89)	0.91 (0.81-0.97)
> 3.0 cm (n = 5)	8	5/5 (100%)	1.00 (0.48-1.00)	0.94 (0.83-0.99)	0.63 (0.36-0.83)	1.00 (0.92-1.00)	16.67 (5.56-49.93)	0.00 (NA)	0.95 (0.85-0.99)
**Nodule echogenicity**									
Hypoechoic (n = 7)	9	6/7 (86%)	0.86 (0.42-1.00	0.94 (0.83-0.99)	0.67 (0.39-0.86)	0.98 (0.88-1.00)	14.29 (4.58-44.58)	0.15 (0.02-0.93)	0.93 (0.83-0.98)
Isoechoic (n = 8)	6	3/8 (38%)	0.38 (0.09-0.76)	0.94 (0.83-0.99)	0.50 (0.20-0.80)	0.90 (0.85-0.94)	6.25 (1.52-25.74)	0.66 (0.39-1.14)	0.86 (0.75-0.94)
Hyperechoic (n = 6)	9	6/6 (100%)	1.00 (0.54-1.00)	0.94 (0.83-0.99)	0.67 (0.40-0.86)	1.00 (0.92-1.00)	16.67 (5.56-49.93)	0.00 (NA)	0.95 (0.85-0.99)
Heterogeneous (n = 4)	6	3/4 (75%)	0.75 (0.19-0.99)	0.94 (0.83-0.99)	0.98 (0.90-1.00)	0.98 (0.90-1.00)	12.5 (3.64-42.96)	0.27 (0.05-1.45)	0.93 (0.82-0.98)
**Reader 2 without deep learning assistance**	23	19/25 (76%)	0.76 (0.55-0.91)	0.92 (0.81-0.98)	0.83 (0.64-0.93)	0.88 (0.79-0.94)	9.50 (3.62-24.95)	0.26 (0.13-0.53)	0.87 (0.77-0.93)
**LI-RADS visualization score**									
VIS-A (n = 31)	11	10/12 (83%)	0.83 (0.52-0.98)	0.95 (0.74-1.00)	0.91 (0.59-0.99)	0.90 (0.72-0.97)	15.83 (2.31-108.48)	0.18 (0.05-0.63)	0.90 (0.74-0.98)
VIS-B (n = 24)	9	7/8 (88%)	0.88 (0.47-1.00)	0.88 (0.62-0.98)	0.78 (0.48-0.93)	0.93 (0.69-0.99)	7.00 (1.87-26.27)	0.14 (0.02-0.90)	0.88 (0.68-0.97)
VIS-C (n = 20)	3	2/5 (40%)	0.40 (0.05-0.85)	0.93 (0.68-1.00)	0.67 (0.18-0.95)	0.82 (0.69-0.91)	6.00 (0.68-52.90)	0.64 (0.31-1.33)	0.80 (0.56-0.94)

**Nodule size**									
≤ 2.0 cm (n = 12)	11	7/12 (58%)	0.58 (0.28-0.85)	0.92 (0.81-0.98)	0.64 (0.38-0.83)	0.90 (0.82-0.95)	7.29 (2.54-20.93)	0.45 (0.23-0.89)	0.85 (0.74-0.93)
2.0-3.0 cm (n = 8)	11	7/8 (88%)	0.88 (0.47-1.00)	0.92 (0.81-0.98)	0.64 (0.40-0.82)	0.98 (0.88-1.00)	10.94 (4.12-29.02)	0.14 (0.02-0.85)	0.91 (0.81-0.97)
> 3.0 cm (n = 5)	9	5/5 (100%)	1.00 (0.48-1.00)	0.92 (0.81-0.98)	0.56 (0.33-0.76)	1.00 (0.92-1.00)	12.5 (4.88-32.00)	0.00 (NA)	0.93 (0.82-0.98)
**Nodule echogenicity**									
Hypoechoic (n = 7)	10	6/7 (86%)	0.86 (0.42-1.00)	0.92 (0.81-0.98)	0.60 (0.36-0.80)	0.98 (0.88-1.00)	10.71 (3.99-28.76)	0.13 (0.03-0.95)	0.91 (0.81-0.97)
Isoechoic (n = 8)	8	4/8 (50%)	0.50 (0.16-0.84)	0.92 (0.81-0.98)	0.50 (0.24-0.76)	0.92 (0.85-0.96)	6.25 (1.94-20.09)	0.54 (0.27-1.09)	0.86 (0.75-0.94)
Hyperechoic (n = 6)	10	6/6 (100%)	1.00 (0.54-1.00)	0.92 (0.81-0.98)	0.60 (0.67-0.79)	1.00 (0.92-1.00)	12.5 (4.88-32.00)	0 (NA)	0.93 (0.83-0.98)
Heterogeneous (n = 4)	7	3/4 (75%)	0.75 (0.19-0.99)	0.92 (0.81-0.98)	0.43 (0.20-0.69)	0.98 (0.89-1.00)	9.38 (3.13-28.08)	0.27 (0.05-1.49)	0.91 (0.80-0.97)
**Reader 2 with deep learning assistance**	27	19/25 (76%)	0.76 (0.55-0.91)	0.84 (0.71-0.93)	0.70 (0.55-0.82)	0.88 (0.78-0.93)	4.75 (2.43-9.30)	0.29 (0.14-0.58)	0.81 (0.71-0.89)
**LI-RADS visualisation score**									
VIS-A (n = 31)	12	9/12 (75%)	0.75 (0.43-0.95)	0.84 (0.60-0.97)	0.75 (0.50-0.90)	0.84 (0.66-0.94)	4.75 (1.6-14.11)	0.3 (0.11-0.81)	0.81 (0.63-0.93)
VIS-B (n = 24)	11	8/8 (100%)	1.00 (0.63-1.00)	0.81 (0.54-0.96)	0.73 (0.49-0.88)	1.00 (0.75-1.00)	5.33 (1.92-14.79)	0.00 (NA)	0.88 (0.68-0.97)
VIS-C (n = 20)	4	2/5 (40%)	0.40 (0.05-0.85)	0.67 (0.60-0.98)	0.50 (0.67-0.90)	0.81 (0.67-0.90)	3.00 (0.56-16.07)	0.69 (0.33-1.45)	0.75 (0.51-0.91)
**Nodule size**									
≤ 2.0 cm (n = 12)	16	8/12 (67%)	0.67 (0.35-0.90)	0.84 (0.71-0.93)	0.50 (0.32-0.68)	0.91 (0.82-0.96)	4.17 (1.97-8.83)	0.4 (0.18-0.89)	0.81 (0.69-0.90)
2.0-3.0 cm (n = 8)	14	6/8 (75%)	0.75 (0.35-0.97)	0.84 (0.71-0.93)	0.43 (0.36-0.61)	0.95 (0.86-0.99)	4.69 (2.21-9.93)	0.3 (0.09-0.99)	0.83 (0.71-0.91)
> 3.0 cm (n = 5)	13	5/5 (100%)	1.00 (0.48-1.00)	0.84 (0.71-0.93)	0.38 (0.25-0.54)	1.00 (0.92-1.00)	6.25 (3.31-11.80)	0 (NA)	0.85 (0.73-0.94)

**Nodule echogenicity**									
Hypoechoic (n = 7)	14	6/7 (86%)	0.86 (0.42-0.91)	0.84 (0.71-0.93)	0.43 (0.27-0.60)	0.98 (0.87-1.00)	5.36 (2.65-10.83)	0.17 (0.03-1.05)	0.84 (0.72-0.93)
Isoechoic (n = 8)	12	4/8 (50%)	0.50 (0.16-0.84)	0.84 (0.71-0.93)	0.33 (0.16-0.56)	0.91 (0.84-0.96)	3.12 (1.22-8.00)	0.6 (0.29-1.20)	0.79 (0.67-0.89)
Hyperechoic (n = 6)	14	6/6 (100%)	1.00 (0.54-1.00)	0.84 (0.71-0.93)	0.43 (0.28-0.59)	1.00 (0.92-1.00)	6.25 (3.31-11.80)	0.00 (NA)	0.86 (0.74-0.94)
Heterogeneous (n = 4)	11	3/4 (75%)	0.75 (0.19-0.99)	0.84 (0.71-0.93)	0.27 (0.174-0.47)	0.98 (0.88-1.00)	4.69 (2.00-10.97)	0.3 (0.05-1.63)	0.83 (0.71-0.92)
**Reader 3 without deep learning assistance**	24	19/25 (76%)	0.76 (0.55-0.91)	0.88 (0.76-0.95)	0.76 (0.59-0.87)	0.88 (0.78-0.94)	6.33 (2.90-13.85)	0.27 (0.13-0.55)	0.84 (0.74-0.91)
**LI-RADS visualisation score**									
VIS-A	13	10/13 (77%)	0.77 (0.46-0.95)	0.86 (0.64-0.97)	0.77 (0.53-0.91)	0.86 (0.69-0.94)	5.38 (1.81-16.00)	0.27 (0.10-0.74)	0.82 (0.65-0.93)
VIS-B	8	7/8 (88%)	0.88 (0.66-1.00)	0.93 (0.66-1.00)	0.88 (0.67-0.99)	0.93 (0.67-0.99)	12.25 (1.82-82.46)	0.13 (0.02-0.85)	0.91 (0.71-0.99)
VIS-C	4	2/4 (50%)	0.50 (0.07-0.93)	0.87 (0.60-0.98)	0.50 (0.17-0.83)	0.87 (0.71-0.95)	3.75 (0.74-18.95)	0.58 (0.21-1.57)	0.79 (0.54-0.94)
**Nodule size**									
≤ 2.0 cm (n = 12)	13	8/12 (67%)	0.67 (0.35-0.90)	0.90 (0.78-0.97)	0.62 (0.39-0.80)	0.92 (0.83-0.96)	6.67 (2.65-16.78)	0.37 (0.17-0.83)	0.85 (0.74-0.93)
2.0-3.0 cm (n = 8)	11	6/8 (75%)	0.75 (0.35-0.97)	0.90 (0.78-0.97)	0.55 (0.32-0.75)	0.96 (0.87-0.99)	7.50 (2.98-18.87)	0.28 (0.08-0.93)	0.88 (0.77-0.95)
> 3.0 cm (n = 5)	10	5/5 (100%)	1.00 (0.48-1.00)	0.90 (0.78-0.97)	0.50 (0.30-0.70)	1.00 (0.92-1.00)	10.00 (4.35-22.97)	0.00 (NA)	0.91 (0.80-0.97)
**Nodule echogenicity**									
Hypoechoic (n = 7)	12	6/7 (86%)	0.86 (0.42-1.00)	0.90 (0.78-0.97)	0.55 (0.33-0.74)	0.98 (0.88-1.00)	8.57 (3.54-20.77)	0.16 (0.03-0.98)	0.89 (0.78-0.96)
Isoechoic (n = 8)	10	4/8 (50%)	0.50 (0.16-0.84)	0.90 (0.78-0.97)	0.44 (0.21-0.70)	0.92 (0.85-0.96)	5.00 (1.69-14.76)	0.56 (0.28-1.12)	0.84 (0.76-0.93)
Hyperechoic (n = 6)	12	6/6 (100%)	1.00 (0.54-1.00)	0.90 (0.78-0.97)	0.55 (0.34-0.63)	1.00 (0.92-1.00)	10.00 (4.35-22.97)	0.00 (NA)	0.91 (0.80-0.97)

Heterogeneous (n = 4)	9	3/4 (75%)	0.75 (0.19-0.99)	0.90 (0.78-0.97)	0.38 (0.18-0.62)	0.98 (0.89-1.00)	7.50 (2.74-20.51)	0.28 (0.05-1.52)	0.89 (0.77-0.96)
**Reader 3 with deep learning assistance**	23	19/25 (76%)	0.76 (0.55-0.91)	0.92 (0.81-0.98)	0.83 (0.64-0.93)	0.88 (0.79-0.94)	9.50 (3.62-24.95)	0.26 (0.13-0.53)	0.87 (0.77-0.93)
**LI-RADS visualisation score**									
VIS-A	13	11/13 (85%)	0.85 (0.55-0.98)	0.90 (0.70-0.99)	0.85 (0.59-0.95)	0.90 (0.72-0.97)	8.88 (2.33-33.88)	0.17 (0.05-0.61)	0.88 (0.73-0.97)
VIS-B	7	6/8 (75%)	0.75 (0.35-0.97)	0.93 (0.66-1.00)	0.86 (0.47-0.98)	0.87 (0.66-0.96)	10.50 (1.52-72.38)	0.27 (0.08-0.90)	0.86 (0.65-0.97)
VIS-C	3	2/4 (50%)	0.50 (0.07-0.93)	0.93 (0.68-1.00)	0.67 (0.19-0.94)	0.88 (0.72-0.95)	7.50 (0.89-63.24)	0.54 (0.20-1.44)	0.84 (0.60-0.97)
**Nodule size**									
≤ 2.0 cm (n = 12)	12	8/12 (67%)	0.67 (0.35-0.90)	0.92 (0.81-0.98)	0.67 (0.42-0.85)	0.92 (0.84-0.96)	8.33 (3.00-23.15)	0.36 (0.16-0.81)	0.87 (0.76-0.94)
2.0-3.0 cm (n = 8)	10	6/8 (75%)	0.75 (0.35-0.97)	0.92 (0.81-0.98)	0.60 (0.35-0.81)	0.96 (0.87-0.99)	9.38 (3.38-26.04)	0.27 (0.08-0.90)	0.90 (0.79-0.96)
> 3.0 cm (n = 5)	9	5/5 (100%)	1.00 (0.48-1.00)	0.92 (0.81-0.98)	0.56 (0.33-0.76)	1.00 (0.92-1.00)	12.50 (4.88-32.00)	0.00 (NA)	0.93 (0.82-.098)
**Nodule echogenicity**									
Hypoechoic (n = 7)	10	6/7 (86%)	0.86 (0.42-1.00)	0.92 (0.81-0.98)	0.60 (0.36-0.80)	0.98 (0.88-1.00)	10.71 (3.99-28.76)	0.16 (0.03-0.95)	0.91 (0.81-0.97)
Isoechoic (n = 8)	8	4/8 (50%)	0.50 (0.16-0.84)	0.92 (0.81-0.98)	0.50 (0.24-0.76)	0.92 (0.85-0.96)	6.25 (1.94-20.09)	0.54 (0.27-1.09)	0.86 (0.75-0.94)
Hyperechoic (n = 6)	10	6/6 (100%)	1.00 (0.54-1.00)	0.92 (0.81-0.98)	0.60 (0.37-0.79)	1.00 (0.92-1.00)	12.50 (4.88-32.00)	0.00 (NA)	0.93 (0.83-0.98)
Heterogeneous (n = 4)	7	3/4 (75%)	0.75 (0.19-0.99)	0.92 (0.81-0.98)	0.43 (0.20-0.69)	0.98 (0.89-1.00)	9.38 (3.13-28.08)	0.27 (0.05-1.49)	0.91 (0.80-0.97)

Table S4 – Subgroup performances of the 3 radiologists without and with the assistance of the deep learning object detection model for detecting early-stage HCC (BCLC 0 or A)
